# Predicting patient-level new-onset atrial fibrillation from population-based nationwide electronic health records: protocol of FIND-AF for developing a precision medicine prediction model using artificial intelligence

**DOI:** 10.1136/bmjopen-2021-052887

**Published:** 2021-11-02

**Authors:** Ramesh Nadarajah, Jianhua Wu, Alejandro F Frangi, David Hogg, Campbell Cowan, Chris Gale

**Affiliations:** 1 Leeds Institute for Data Analytics, University of Leeds, Leeds, UK; 2 Leeds Institute of Cardiovascular and Metabolic Medicine, University of Leeds, Leeds, UK; 3 Department of Cardiology, Leeds Teaching Hospitals NHS Trust, Leeds, UK; 4 School of Dentistry, University of Leeds, Leeds, UK; 5 School of Computing, University of Leeds, Leeds, UK

**Keywords:** primary care, pacing & electrophysiology, adult cardiology, cardiac epidemiology, health informatics

## Abstract

**Introduction:**

Atrial fibrillation (AF) is a major cardiovascular health problem: it is common, chronic and incurs substantial healthcare expenditure because of stroke. Oral anticoagulation reduces the risk of thromboembolic stroke in those at higher risk; but for a number of patients, stroke is the first manifestation of undetected AF. There is a rationale for the early diagnosis of AF, before the first complication occurs, but population-based screening is not recommended. Previous prediction models have been limited by their data sources and methodologies. An accurate model that uses existing routinely collected data is needed to inform clinicians of patient-level risk of AF, inform national screening policy and highlight predictors that may be amenable to primary prevention.

**Methods and analysis:**

We will investigate the application of a range of deep learning techniques, including an adapted convolutional neural network, recurrent neural network and Transformer, on routinely collected primary care data to create a personalised model predicting the risk of new-onset AF over a range of time periods. The Clinical Practice Research Datalink (CPRD)-GOLD dataset will be used for derivation, and the CPRD-AURUM dataset will be used for external geographical validation. Both comprise a sizeable representative population and are linked at patient-level to secondary care databases. The performance of the deep learning models will be compared against classic machine learning and traditional statistical predictive modelling methods. We will only use risk factors accessible in primary care and endow the model with the ability to update risk prediction as it is presented with new data, to make the model more useful in clinical practice.

**Ethics and dissemination:**

Permissions for CPRD-GOLD and CPRD-AURUM datasets were obtained from CPRD (ref no: 19_076). The CPRD ethical approval committee approved the study. The results will be submitted as a research paper for publication to a peer-reviewed journal and presented at peer-reviewed conferences.

**Trial registration details:**

A systematic review to incorporate within the overall project was registered on PROSPERO (registration number CRD42021245093). The study was registered on ClinicalTrials.gov (NCT04657900).

**Strengths and limitations of this study**

- Large and nationwide dataset representative of the UK primary care population.
- Using artificial intelligence technology may discover new predictive variables by efficiently incorporating temporal information of event data.
- The model will not just predict the risk of a patient developing atrial fibrillation, but also provide a representation of how risk develops over time to enable more focused screening.
- The derivation and validation work will be undertaken in datasets collected over the same time period in the UK; therefore, further validation work may be pursued with newly collected data and for international contexts.
- The derivation data will not include unstructured natural language free text; future research could explore if incorporating free text into representation learning improves predictive accuracy.

## Introduction

Atrial fibrillation (AF) is the most common sustained cardiac arrhythmia. The current estimated prevalence is between 2% and 4%, and a 2.3-fold rise is expected due to extended longevity in the general population.^1^ While AF may present with symptomatic palpitations, for many patients, the first diagnosis of AF is only after they present with stroke or cardiac decompensation. The frequency of AF in ischaemic strokes is 20%–30%, and these are usually severe, resulting in permanent disability or fatality.^2^

Oral anticoagulants can reduce the risk of stroke by up to two-thirds in those with AF at higher risk of stroke.^3^ International guidelines recommend that patients with AF at elevated thromboembolic risk are offered stroke prophylaxis with an oral anticoagulant.^1^ Most patients with AF will have stroke risk factors, making them eligible for an oral anticoagulant. Many will have concomitant cardiovascular disease (such as hypertension, valvular heart disease or heart failure) making them eligible for further investigation or treatment. Equally, in those with AF who are at low risk of stroke (and therefore do not qualify for oral anticoagulation), surveillance for increasing stroke risk is advisable.

Thus, the early diagnosis of AF, before the manifestation of the first complication, remains a major public health challenge. Screening for AF in the community has been proposed as an approach to optimise early AF detection.^4^ Previous studies have shown that the risk of AF (often asymptomatic) increases with age.^5^ Opportunistic screening is cost-effective in patients aged 65 years or over, and among individuals aged 75–76 years old undergoing a 2-week intermittent ECG screening.^6–8^ Nevertheless, there is no current recommendation for UK population-wide systematic screening.^9^

Prediction models could contribute to AF screening by discriminating patients into risk categories, from which investigation intensity could be planned.^10^ However, models based solely on analysis of investigations may not apply in the community setting—for example, routine ECGs are not always available.^11^

To date, several multivariable prediction models have been created or tested for prediction of incident AF in the community. The earliest models were derived from structured follow-up of prospective cohorts, including Framingham Heart Study score for Atrial Fibrillation and CHARGE-AF (Cohorts for Heart and Ageing Research in Genomic Epidemiology for Atrial Fibrillation).^12 13^ The proliferation of electronic health records (EHRs) has led to the development of several models from local registries, including Maccabi Healthcare Services and C_2_HEST (Coronary artery disease/chronic obstructive pulmonary disease, Hypertension, Elderly, Systolic heart failure, Thyroid disease).^14 15^ Structured EHRs offer larger sample sizes to assess more candidate variables and derive more generalisable models.

A systematic review found that the models derived from prospective cohorts had been more frequently externally validated. CHARGE-AF was the only model that showed significant overall discrimination in meta-analysis but its transportability to EHRs is still being investigated.^16 17^ Each model to date is, however, limited by one or more of their use of small, geographically remote or historical datasets, lack of temporal information, crude risk modelling with consequent suboptimal model performance and/or predictor variables not readily available in primary care.^18^ None has yet reached widespread clinical practice.

Machine learning is a data-driven approach that can identify non-linear associations and complex interactions between variables without the need to specify these relationships *a priori*.^19^ A recent study applying this methodology to a nationwide UK dataset produced a model with a greater discriminative capability than CHARGE-AF (area under receiver operating characteristic (AUC) 0.827 vs 0.725) in EHR.^19^

Artificial intelligence (AI) has several desirable features for prediction modelling from EHRs. It facilitates the use of vast quantities of event data and associated temporal information, handles many predictors with automatic variable selection techniques, accommodates non-linearities and interactions among variables, and enables a live learning approach (whereby the prediction model is automatically updated). A range of AI techniques have been applied to EHR data and have demonstrated better prediction power over traditional statistical approaches.^20^ Furthermore deep learning, a subfield of machine learning, can learn complex patterns from data to characterise higher level correlations among clinical events.^21^ Accordingly, it may derive robust patient representations from raw EHR data for prediction modelling without the need for manual, expert-dependent feature engineering like classic machine learning techniques, which places a limit on scalability and generalisability.^22^

Using AI to develop a predictive algorithm from routinely collected primary care EHRs could offer several advantages:

1. A model could predict the risk that a person will develop new-onset AF, and how that evolves over time, whereas current prediction models only provide a fixed prediction horizon. This would allow phenotype-specific and temporal-specific screening which could make screening more efficacious and cost-effective.
2. A model created from routinely collected EHRs could be more smoothly translated into clinical practice by being embedded into existing clinical EHRs.
3. The predictive magnitude of variables for the development of AF may identify novel risk markers for AF, which could then be studied for causality.

### Research aim

The aims of the Future Innovations in Novel Detection of Atrial Fibrillation (FIND-AF) study are to:

1. Develop a deep learning model for predicting the risk, *and evolution of the risk,* of new-onset AF in primary care.
2. Identify and quantify the magnitude of risk markers of new AF among routinely collected primary care data.
3. Externally validate the model in a geographically distinct dataset to assess generalisability.

## Methods and analysis

### Data sources and permissions

The derivation dataset for training and testing the model will be the Clinical Practice Research Datalink-GOLD (CPRD-GOLD) dataset. This is an ongoing primary care database, established in 1987, that comprises anonymised medical records and prescribing data contributed by general practices using Vision software. It contains data for approximately 17.5 million patients, with 30% of contributing practices in England, and represents the UK population in terms of age, sex and ethnicity.^23^ In order to contribute to the database, general practices and other health centres must meet prespecified standards for research-quality data (‘up-to-standard’).^23 24^

To ascertain whether the prediction model is generalisable, we will externally validate its performance in the geographically distinct CPRD-AURUM dataset. This was launched in 2017 and encompasses only practices using EMIS Web software. It contains data for approximately 26.9 million patients and draws on data collected from practices in England only.^25^ Any practices which previously contributed to CPRD-GOLD have been removed from the CPRD-GOLD cohort to ensure that these datasets reflect different populations. CPRD undertakes various levels of validation and quality assurance on the daily general ptactice data collection comprising over 900 checks covering the integrity, structure and format of the data.^25^

Recorded information in both datasets includes patients’ demography, clinical symptoms, signs, investigations, diagnoses, prescriptions, referrals, behavioural factors and test results entered by clinicians and other practice staff. All clinical information is coded using Read Codes in CPRD-GOLD and SNOMED clinical terms (CT) in CPRD-AURUM.^26 27^ In the proposed study, extracted patients will have patient-level data linked to Hospital Episode Statistics (HES) Admitted Patient Care (APC) and Diagnostic Imaging Dataset (DID), Office for National Statistics (ONS) Death Registration, patient-level deprivation and practice-level deprivation to provide a more comprehensive dataset. The CPRD dataset has been used to develop or validate a range of risk prediction models, including in cardiovascular disease.^19 28^

The extracted datasets, including linked data, comprise all patients for the period between 2 January 1998 and 30 November 2018 from the snapshot of CPRD-GOLD and CPRD-AURUM provided in October 2019. Over this study period, the CPRD-GOLD dataset comprises approximately 4.5 million patients eligible for data linkage at an up-to-standard practice, with over 200 000 patients having a record of AF. The CPRD-AURUM dataset comprises approximately 18 million patients eligible for data linkage, with almost 800 000 patients having a record of AF. A sample of 245 general practices will be randomly selected from 800 general practices in CPRD-AURUM to approximately match the size of CPRD-GOLD.

### Patient and public involvement

Patients and the public were not involved in the design of this research. However, a Scientific Advisory Board, including representatives from the Arrhythmia Alliance, National Institute for Health and Care Excellence AF guideline committee lay members (last updated 04 January 2020) and EHR software providers, has been created to provide expert context advice on the research, the dissemination of results and advise on the translation of the findings of this study into clinical practice.

### Inclusion and exclusion criteria

The study population will comprise all available patients in CPRD-GOLD and CPRD-AURUM eligible for data linkage and with at least 1-year follow-up in the period between 2 January 1998 and 30 November 2018. Patients will be excluded if they were 18 years of age or under at the date of the first registration in CPRD, diagnosed with AF or atrial flutter (AFl) before 1 January 1998, registered for less than 1 year in CPRD or ineligible for data linkage.

### Outcome ascertainment

The outcome of interest is first diagnosed AF or AFl after baseline (1 January 2009). We have included AFl as an outcome since it has similar clinical relevance, including thromboembolic risk and anticoagulation guidelines, as AF.^1^ These will be identified using Read codes and SNOMED CT in CPRD datasets. For HES APC events and underlying cause of death variable in the ONS Death Registration data file, the 10th revision of the International Statistical Classification of Diseases and Related Health Problems (ICD-10) codes will be used. Misclassified data can lead to systematic prediction errors and accuracy of data may vary over time.^29^ CPRD has converted older ICD codes to the newer version, increasing confidence in their validity. Nonetheless, to verify data accuracy, we will check the data accuracy by year and include the year of AF diagnosis in the prediction models to assess their impact.

### Sample size

To develop a prognostic prediction model, the required sample size may be determined by three criteria suggested by Riley *et al*.^30^ For example, suppose a maximum of 200 parameters will be included in the prediction model and the Cox-Snell generalised R^2^ is assumed to be 0.01. A total of 377 996 patients will be required to meet Riley’s criterion (1) with global shrinkage factor of 0.95; this sample size also ensures a small absolute difference (Δ<0.05) in the apparent and adjusted Nagelkerke R^2^ (Riley’s criterion (2)) and ensures precise estimate of overall risk with a margin of error <0.001 (Riley’s criterion (3)). According to the Quality and Outcomes Framework, the prevalence of AF in England is 1.7%.^31 32^ Given an AF prevalence of 1.7%, only 6425 patients will be expected to develop AF from 377 996 patients. Therefore, the number of patients in the CPRD dataset with AF will provide sufficient statistical power to develop and validate a deep learning prediction algorithm with the predefined precision and accuracy.

### Predictor variables

A systematic review has highlighted 22 predictor variables included in varying combinations by 10 preceding prediction models developed to detect incident AF in the community.^16^ In addition, a more recently published machine learning model has established six further time-varying variables (eg, change in body mass index between the latest two quarters).^19^

To capture the full potential of deep learning and this large dataset, we will broaden our search for candidate predictors to all available variables, while retaining temporal information (including all clinical assessments, hospitalised events and medications). The potential predictors may include the following:

1. Sociodemographic variables including age, sex, ethnicity and indices of multiple deprivation.
2. All disease conditions during follow-up, including hospitalised diseases and procedures, such as other cardiovascular diseases, diabetes mellitus, chronic lung disease, renal disease, inflammatory disease, cancer, hypothyroidism and surgical procedures.
3. Clinical assessments including heart rate, systolic and diastolic blood pressure, height, weight and body mass index.
4. Medications prescribed including antihypertensives, statins, antidepressants, anxiolytics/hypnotics and antipsychotics.
5. Lifestyle factors including smoking status and alcohol consumption.
6. All biomarkers routinely collected during follow-up including total, high-density lipoprotein and low-density lipoprotein cholesterol, triglycerides, creatinine, C reactive protein, erythrocyte sedimentation rate.

Predictive factors will be identified using the appropriate codes. In CPRD, we will use Read codes for diagnoses, measurements (eg, systolic and diastolic values) and product codes (prodcodes) for medications. In HES APC, we will use ICD-10 codes and Office of Population Censuses and Surveys Classification of Interventions and Procedures version 4 (OPCS-4) codes. In the ONS Death Registration data file, we will use ICD-10 codes (and ICD-9 codes for the period before 2001).

### Missing data

Missing data are expected in EHR data, and will be handled using multiple imputations according to the approaches suggested by Carpenter and Kenward, and depending on the amount of missing data.^33^ It is likely there will be misclassification of baseline characteristics, such as smoking status, cholesterol, blood pressure and weight that change over time. To account for this, we will define baseline information using only measures recorded within the last year. We will then use measures taken within the last 2–5 years and in the year after baseline as parameters in the chained imputation equations used to impute baseline covariates with ≤40% missingness. In addition, median imputation, a common approach to deal with missing values in machine learning algorithms, will be used to test the model robustness.^34^

### Data analysis plan

#### Development and external validation of model

The CPRD-GOLD and CPRD-AURUM data will be cleaned and preprocessed for model development and validation, respectively. Specifically, for patient features with binary values, 0 and 1 will be mapped to the binary values. Variables with multiple categories will be split into their component categories, and each given a binary value to indicate the presence or not of the variable for each patient. Continuous variables will be kept as continuous. To reduce the high cardinality of Read codes and ICD codes, we will map both to Caliber codes,^35^ which is an expert checked mapping directory from University College London. Prodcodes can be mapped to level 2 of British National Formulary codes.^36^

A number of deep learning models will be investigated for prediction of AF in CPRD-GOLD. These will include a convolutional neural network (CNN), a recurrent neural network (RNN) and a Transformer architecture. They each possess characteristics that can capture the temporality of EHR data and the progression of a patient’s health status.

**Figure 1:**
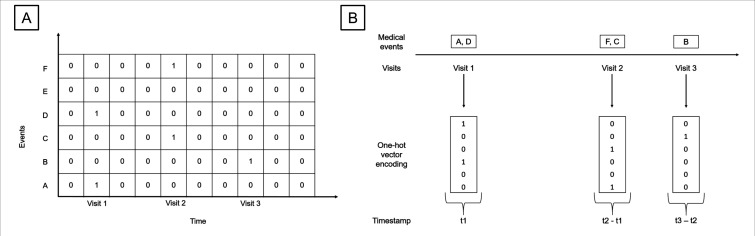
An example of how a patient’s EHR could be represented as a temporal matrix (A) compared with a sequence (B). In (A), time is on the x dimension and medical events are on the y dimension. In (B), the temporal information, in this example, is represented as intervisit interval through timestamps (eg, t2–t1). EHR, electronic health record.

Although CNNs are typically associated with static content (eg, images and documents), they are increasingly used to uncover temporal relationships in EHR.^37–40^ The patient EHR can be converted to a temporal matrix, with one dimension corresponding to time and the other dimension corresponding to medical events (figure 1A).^37^ A one-sided convolution operation can be applied to each possible window of features in the event matrix to produce a feature map followed with max pooling (to capture the most important features) and culminating in a fully connected layer and softmax classifier. If the time dimension is embedded in 1-day increments, we have the option to learn temporal features by extending connectivity in the time dimension through a range of fusion strategies. Temporal early fusion could combine information across an entire time window to establish global patterns, temporal late fusion can be performed in the fully connected layer to model local connections and temporal slow fusion could capture both by extending the connectivity of all convolutional layers.^37^

Patient EHR events including diagnoses, procedures and medications can also be represented as a sequence of codes over time, similar to words in a sentence (figure 1B). After embedding into a lower dimensional space, a one-dimensional convolutional operation over the temporal dimension with a combination of filters of different lengths could capture temporal dependencies at multiple levels.^38^

A sequence-based representation also makes patient EHR amenable to techniques that have provided breakthroughs in natural language processing, especially RNNs.^41–43^ RNNs with a gated recurrent unit model design have been successfully applied in modelling sequential structured EHR data to predict diagnoses as they can accept an input vector at each time step while storing information in a hidden layer which changes over time.^21 44–51^ We will apply the reverse time attention model (RETAIN), which has been tested for the prediction of heart failure in CPRD.^52^ RETAIN can generate temporal attention vectors at both visit and variable level by running RNNs backwards, which somewhat mimics a physician’s behaviours in examining a patient’s past visits in reverse time order and could capture the short-term time-varying events that were found to improve prediction in a previous shallow neural network model.^19 46^ Timestamps can be included to calculate the attentions for the entire visit sequence and thus provide temporal information for each visit.^46^

Finally, Transformers have become state of the art in natural language processing,^53 54^ and we will apply an adaption for EHR, BEHRT (BERT [Bidirectional Encoder Representations from Transformers] for EHR), which was originally developed in CPRD.^55^ This model’s structure, depicting each diagnosis code as a word, with each visit as a sentence and the entire medical history as a document, facilitates multihead self-attention, positional encoding and masked language model for EHR. BEHRT can define a representation incorporating information on the diseases afflicting a patient, the positional interactions of diseases and sequence of events, with the age of a patient linked to each visit to give a sense of time between diagnoses.^55^ This representation can then be used for a number of tasks including disease prediction.

Preprocessed patient-level data in CPRD-GOLD will be randomly split into an 80:20 ratio to create training and testing samples. The split ratio is not a significant factor, given the volume of the sample size. The model parameters and dropout rate will be chosen through a grid search and 10-fold cross-validation will be used (ie, 10% of the training data will be randomly selected as the cross-validation set).

While the outcome is binary, instead of just predicting 0 or 1 for a patient (new AF or not), the probability of that patient developing AF by different time points over months (1, 3, 6) and years (1, 5, 10) may be predicted. The probability of developing AF at each time point could be plotted to give an understanding of evolving risk of AF. The clinical risk prediction performance of the deep learning models will be compared against a range of classic machine learning techniques and traditional statistical predictive modelling methods including support vector machine, random forest, naïve Bayes and Cox proportional hazards model. Discrimination (Harrell’s c-statistic and AUC) and calibration metrics will be supplemented with positive and negative predictive values, precision, recall and area under precision-recall curve for all models.

The CPRD-AURUM dataset will then externally validate the model performance to assess generalisability. It has been shown that a lack of external validation has hampered the implementation of previous machine learning models in routine clinical practice.^56^

#### Identification and quantification of the magnitude of predictors

The proposed deep learning models can extract informative risk factors from EHR data. Specifically, a risk factor selection strategy proposed by Huang *et al*^57^ will be adapted to identify informative risk factors. The model will provide weights of the identified risk factors to help understand the significance of risk factors at different risk levels. The impact of the number of risk factors on AF risk prediction performance will be assessed through the curves of both AUC and prediction accuracy plotted against the number of risk factors. Some predictors, such as body mass index, blood pressure, frequency of visits and strength of the prescribed medication, may change over time. The incremental prognostic values of including these variable trajectories will be explored, and the impact on predictive accuracy will be assessed.

### Software

The deep learning model will be implemented in R (through the R studio interface) and TensorFlow or Python and PyTorch including data preprocessing, missing data imputation, model development and validation.

### Ethics and dissemination

The study has been approved by CPRD (ref no: 19_076). Those handling data have completed University of Leeds information security training. All analyses will be conducted in concordance with the CPRD study dataset agreement between the Secretary of State for Health and Social Care and the University of Leeds.

The study is informed by the Prognosis Research Strategy (PROGRESS) framework and recommendations.^58^ The subsequent research paper will be submitted for publication in a peer-reviewed journal and will be written following Transparent Reporting of a multivariable prediction model for Individual Prognosis or Diagnosis (TRIPOD) and REporting of studies Conducted using Observational Routinely collected health Data (RECORD) guidelines.^59 60^

If the model succeeds (defined as improving predicting accuracy by at least 5% compared with existing models), the algorithm could be made readily available through free-to-use software. The model will be designed to be amenable to in situ updating with new information so that prediction of an individual’s AF risk is updated contemporaneously. The algorithm could be a built-in tool for use in general practices to ‘screen’ for patients at high risk of developing new-onset AF. Future research will be needed to assess the clinical impact of this risk model. At the point when utilisation in clinical practice is possible, the applicable regulation on medicine devices will be adhered to.^61^ When in clinical use, the model itself could also be reviewed and updated by a prespecified expert consensus group on an annual basis after incorporating evidence from post-service utilisation and the curation of more data.

## Conclusions

AF is a common clinical problem with potentially catastrophic sequelae. A prediction model that may identify in a community setting which individuals will develop AF, and when this is most likely to occur, could enable targeted screening. This British Heart Foundation-funded study will contribute to knowledge about the detection of AF through prediction using a data science approach in routine EHR data. The use of AI technology may uncover new predictors in EHR and facilitate easier translation into clinical practice.

## Supplementary Material

Author's manuscript

## References

[1] Hindricks G, Potpara T, Dagres N, et al. 2020 ESC guidelines for the diagnosis and management of atrial fibrillation developed in collaboration with the European association for Cardio-Thoracic surgery (EACTS). Eur Heart J 2021;42:373–498. 10.1093/eurheartj/ehaa612 32860505

[2] Benjamin EJ, Virani SS, Callaway CW, et al. Heart disease and stroke statistics-2018 update: a report from the American heart association. Circulation 2018;137:e67–492. 10.1161/CIR.0000000000000558 29386200

[3] Ruff CT, Giugliano RP, Braunwald E, et al. Comparison of the efficacy and safety of new oral anticoagulants with warfarin in patients with atrial fibrillation: a meta-analysis of randomised trials. Lancet 2014;383:955–62. 10.1016/S0140-6736(13)62343-0 24315724

[4] Freedman B, Camm J, Calkins H, et al. Screening for atrial fibrillation: a report of the AF-SCREEN International Collaboration. Circulation 2017;135:1851–67. 10.1161/CIRCULATIONAHA.116.026693 28483832

[5] Boriani G, Laroche C, Diemberger I, et al. Asymptomatic atrial fibrillation: clinical correlates, management, and outcomes in the EORP-AF pilot General registry. Am J Med 2015;128:509–18. 10.1016/j.amjmed.2014.11.026 25534423

[6] Aronsson M, Svennberg E, Rosenqvist M, et al. Cost-Effectiveness of mass screening for untreated atrial fibrillation using intermittent ECG recording. Europace 2015;17:1023–9. 10.1093/europace/euv083 25868469

[7] Hobbs FDR, Fitzmaurice DA, Mant J, et al. A randomised controlled trial and cost-effectiveness study of systematic screening (targeted and total population screening) versus routine practice for the detection of atrial fibrillation in people aged 65 and over. The SAFE study. Health Technol Assess 2005;9:iii-iv, ix-x, 1-74. 10.3310/hta9400 16202350

[8] Svennberg E, Engdahl J, Al-Khalili F, et al. Mass screening for untreated atrial fibrillation: the STROKESTOP study. Circulation 2015;131:2176–84. 10.1161/CIRCULATIONAHA.114.014343 25910800

[9] Committee UNS. The UK NSC recommendation on atrial fibrillation screening in adults, 2019. Available: https://legacyscreening.phe.org.uk/atrialfibrillation#:~:text=The%20UK%20NSC%20does%20not,in%20people%20found%20through%20screening

[10] Moons KGM, Kengne AP, Woodward M, et al. Risk prediction models: I. Development, internal validation, and assessing the incremental value of a new (bio)marker. Heart 2012;98:683–90. 10.1136/heartjnl-2011-301246 22397945

[11] Attia ZI, Noseworthy PA, Lopez-Jimenez F, et al. An artificial intelligence-enabled ECG algorithm for the identification of patients with atrial fibrillation during sinus rhythm: a retrospective analysis of outcome prediction. Lancet 2019;394:861–7. 10.1016/S0140-6736(19)31721-0 31378392

[12] Alonso A, Krijthe BP, Aspelund T, et al. Simple risk model predicts incidence of atrial fibrillation in a racially and geographically diverse population: the CHARGE-AF Consortium. J Am Heart Assoc 2013;2:e000102. 10.1161/JAHA.112.000102 23537808 PMC3647274

[13] Schnabel RB, Sullivan LM, Levy D, et al. Development of a risk score for atrial fibrillation (Framingham heart study): a community-based cohort study. Lancet 2009;373:739–45. 10.1016/S0140-6736(09)60443-8 19249635 PMC2764235

[14] Aronson D, Shalev V, Katz R, et al. Risk score for prediction of 10-year atrial fibrillation: a community-based study. Thromb Haemost 2018;118:1556–63. 10.1055/s-0038-1668522 30103243

[15] Li Y-G, Pastori D, Farcomeni A, et al. A Simple Clinical Risk Score (C_2_HEST) for Predicting Incident Atrial Fibrillation in Asian Subjects: Derivation in 471,446 Chinese Subjects, With Internal Validation and External Application in 451,199 Korean Subjects. Chest 2019;155:510–8. 10.1016/j.chest.2018.09.011 30292759 PMC6437029

[16] Himmelreich JCL, Veelers L, Lucassen WAM, et al. Prediction models for atrial fibrillation applicable in the community: a systematic review and meta-analysis. Europace 2020;22:684–94. 10.1093/europace/euaa005 32011689 PMC7526764

[17] Himmelreich JC, Lucassen WA, Harskamp RE. CHARGE-AF in a national routine primary care electronic health records database in the Netherlands: validation for 5-year risk of atrial fibrillation and implications for patient selection in atrial fibrillation screening 2021;8:e001459. 10.1136/openhrt-2020-001459 PMC7816907 33462107

[18] Kolek MJ, Graves AJ, Xu M, et al. Evaluation of a prediction model for the development of atrial fibrillation in a Repository of electronic medical records. JAMA Cardiol 2016;1:1007–13. 10.1001/jamacardio.2016.3366 27732699 PMC5293184

[19] Hill NR, Ayoubkhani D, McEwan P, et al. Predicting atrial fibrillation in primary care using machine learning. PLoS One 2019;14:e0224582. 10.1371/journal.pone.0224582 31675367 PMC6824570

[20] Weng SF, Reps J, Kai J, et al. Can machine-learning improve cardiovascular risk prediction using routine clinical data? PLoS One 2017;12:e0174944. 10.1371/journal.pone.0174944 28376093 PMC5380334

[21] . Health-atm: a deep architecture for multifaceted patient health record representation and risk prediction. Proceedings of the 2018 SIAM International Conference on Data Mining. SIAM, 2018.

[22] Chen D, Liu S, Kingsbury P, et al. Deep learning and alternative learning strategies for retrospective real-world clinical data. NPJ Digit Med 2019;2:1–5. 10.1038/s41746-019-0122-0 31304389 PMC6550223

[23] Herrett E, Gallagher AM, Bhaskaran K, et al. Data resource profile: clinical practice research Datalink (CPRD). Int J Epidemiol 2015;44:827–36. 10.1093/ije/dyv098 26050254 PMC4521131

[24] Herrett E, Thomas SL, Schoonen WM, et al. Validation and validity of diagnoses in the general practice research database: a systematic review. Br J Clin Pharmacol 2010;69:4–14. 10.1111/j.1365-2125.2009.03537.x 20078607 PMC2805870

[25] Wolf A, Dedman D, Campbell J, et al. Data resource profile: clinical practice research Datalink (cprd) aurum. Int J Epidemiol 2019;48:1740–1740g. 10.1093/ije/dyz034 30859197 PMC6929522

[26] Chisholm J. The read clinical classification. BMJ 1990;300:1092. 10.1136/bmj.300.6732.1092 2344534 PMC1662793

[27] American Medical Informatics Association. SNOMED clinical terms: overview of the development process and project status. Proc AMIA Symp, 2001. PMC2243297 11825268

[28] Hippisley-Cox J, Coupland C, Brindle P. Development and validation of QRISK3 risk prediction algorithms to estimate future risk of cardiovascular disease: prospective cohort study. BMJ 2017;357:j2099. 10.1136/bmj.j2099 28536104 PMC5441081

[29] Ehrenstein V, Nielsen H, Pedersen AB, et al. Clinical epidemiology in the era of big data: new opportunities, familiar challenges. Clin Epidemiol 2017;9:245–50. 10.2147/CLEP.S129779 28490904 PMC5413488

[30] Riley RD, Snell KI, Ensor J, et al. Minimum sample size for developing a multivariable prediction model: PART II - binary and time-to-event outcomes. Stat Med 2019;38:1276–96. 10.1002/sim.7992 30357870 PMC6519266

[31] Cowan JC, Wu J, Hall M, et al. A 10 year study of hospitalized atrial fibrillation-related stroke in England and its association with uptake of oral anticoagulation. Eur Heart J 2018;39:2975–83. 10.1093/eurheartj/ehy411 29982405 PMC6110195

[32] Wu J, Alsaeed ES, Barrett J, et al. Prescription of oral anticoagulants and antiplatelets for stroke prophylaxis in atrial fibrillation: nationwide time series ecological analysis. Europace 2020;22:1311–9. 10.1093/europace/euaa126 32778878 PMC7478320

[33] Carpenter J, Kenward M. Multiple imputation and its application. John Wiley & Sons, 2012.

[34] Batista GEAPA, Monard MC. An analysis of four missing data treatment methods for supervised learning. Applied Artificial Intelligence 2003;17:519–33. 10.1080/713827181

[35] Kuan V, Denaxas S, Gonzalez-Izquierdo A, et al. A chronological map of 308 physical and mental health conditions from 4 million individuals in the English National health service. Lancet Digit Health 2019;1:e63–77. 10.1016/S2589-7500(19)30012-3 31650125 PMC6798263

[36] BNF publications. Available: https://www.bnf.org/ [Accessed 22 Apr 2021].

[37] . Risk prediction with electronic health records: a deep learning approach. Proceedings of the 2016 SIAM International Conference on Data Mining, 2016.

[38] Che Z, Cheng Y, Sun Z. Exploiting convolutional neural network for risk prediction with medical feature embedding 2017.

[39] Wang Y-H, Nguyen P-A, Islam MM, et al. Development of deep learning algorithm for detection of colorectal cancer in EHR data. Stud Health Technol Inform 2019;264:438–41. 10.3233/SHTI190259 31437961

[40] Suo Q, Ma F, Yuan Y, et al. Deep patient similarity learning for personalized healthcare. IEEE Trans Nanobioscience 2018;17:219–27. 10.1109/TNB.2018.2837622 29994534

[41] Mikolov T, Sutskever I, Chen K. Distributed representations of words and phrases and their compositionality 2013.

[42] Zaremba W, Sutskever I, OJapa V. Recurrent neural network regularization 2014.

[43] Shickel B, Tighe PJ, Bihorac A, et al. Deep EHR: a survey of recent advances in deep learning techniques for electronic health record (EHR) analysis. IEEE J Biomed Health Inform 2018;22:1589–604. 10.1109/JBHI.2017.2767063 29989977 PMC6043423

[44] PMLR. Doctor AI: predicting clinical events via recurrent neural networks. Machine learning for healthcare conference, 2016. PMC5341604 28286600

[45] Choi E, Schuetz A, Stewart WF, et al. Using recurrent neural network models for early detection of heart failure onset. J Am Med Inform Assoc 2017;24:361–70. 10.1093/jamia/ocw112 27521897 PMC5391725

[46] Choi E, Bahadori MT, Kulas JA. Retain: an interpretable predictive model for healthcare using reverse time attention mechanism 2016.

[47] . GRAM: graph-based attention model for healthcare representation learning. Proceedings of the 23rd ACM SIGKDD international conference on knowledge discovery and data mining, 2017. 10.1145/3097983.3098126 PMC7954122 33717639

[48] . Dipole: diagnosis prediction in healthcare via attention-based bidirectional recurrent neural networks. Proceedings of the 23rd ACM SIGKDD international conference on knowledge discovery and data mining, 2017.

[49] Kwon BC, Choi M-J, Kim JT. Retainvis: visual analytics with interpretable and interactive recurrent neural networks on electronic medical records 2018;25:299–309. 10.1109/TVCG.2018.2865027 30136973

[50] . Kame: knowledge-based attention model for diagnosis prediction in healthcare. Proceedings of the 27th ACM International Conference on Information and Knowledge Management, 2018.

[51] Choi E, Xiao C, Stewart WF. Mime: multilevel medical embedding of electronic health records for predictive healthcare 2018.

[52] Ayala Solares JR, Diletta Raimondi FE, Zhu Y, et al. Deep learning for electronic health records: a comparative review of multiple deep neural architectures. J Biomed Inform 2020;101:103337. 10.1016/j.jbi.2019.103337 31916973

[53] Devlin J, Chang M-W, Lee K. Bert: Pre-training of deep bidirectional transformers for language understanding 2018.

[54] Vaswani A, Shazeer N, Parmar N. Attention is all you need 2017.

[55] Li Y, Rao S, Solares JRA. BEHRT: transformer for electronic health records 2020;10:1–12. 10.1038/s41598-020-62922-y PMC7189231 32346050

[56] Banerjee A, Chen S, Fatemifar G. Machine learning for subtype definition and risk prediction in heart failure acute coronary syndromes and atrial fibrillation: systematic review of validity and clinical utility 2021;19:1–14. 10.1186/s12916-021-01940-7 PMC8022365 33820530

[57] Huang Z, Dong W, Duan H, et al. A regularized deep learning approach for clinical risk prediction of acute coronary syndrome using electronic health records. IEEE Trans Biomed Eng 2018;65:956–68. 10.1109/TBME.2017.2731158 28742027

[58] Steyerberg EW, Moons KGM, van der Windt DA, et al. Prognosis research strategy (progress) 3: prognostic model research. PLoS Med 2013;10:e1001381. 10.1371/journal.pmed.1001381 23393430 PMC3564751

[59] Collins GS, Reitsma JB, Altman DG, et al. Transparent reporting of a multivariable prediction model for individual prognosis or diagnosis (TRIPOD): the TRIPOD statement. The TRIPOD group. Circulation 2015;131:211–9. 10.1161/CIRCULATIONAHA.114.014508 25561516 PMC4297220

[60] Nicholls SG, Quach P, von Elm E, et al. The reporting of studies conducted using observational routinely-collected health data (record) statement: methods for arriving at consensus and developing reporting guidelines. PLoS One 2015;10:e0125620. 10.1371/journal.pone.0125620 25965407 PMC4428635

[61] Kramer DB, Xu S, Kesselheim AS. Regulation of medical devices in the United States and European Union. The ethical challenges of emerging medical technologies. Taylor and Francis, 2020: 41–9. 10.1056/NEJMhle1113918 22332952

