# Supplementary material for: Predicting patient-level new-onset atrial fibrillation from population-based nationwide electronic health records: protocol of FIND-AF for developing a precision medicine prediction model using artificial intelligence
Source: BMJ Open. 2021 Nov 2;11(11):e052887. doi: 10.1136/bmjopen-2021-052887 (PMC8565546; doi:10.1136/bmjopen-2021-052887)
Supplement: Author's manuscript [file bmjopen-2021-052887.draft_revisions.pdf]

BMJ Open

BMJ Open is committed to open peer review. As part of this commitment we make the peer review history of every article we publish publicly available.

When an article is published we post the peer reviewers' comments and the authors' responses online. We also post the versions of the paper that were used during peer review. These are the versions that the peer review comments apply to.

The versions of the paper that follow are the versions that were submitted during the peer review process. They are not the versions of record or the final published versions. They should not be cited or distributed as the published version of this manuscript.

BMJ Open is an open access journal and the full, final, typeset and author-corrected version of record of the manuscript is available on our site with no access controls, subscription charges or pay-per-view fees (http://bmjopen.bmj.com).

If you have any questions on BMJ Open's open peer review process please email info.bmjopen@bmj.com

BMJ Open

Predicting patient-level new-onset atrial fibrillation from population-based nationwide electronic health records: protocol of FIND-AF for developing a precision medicine prediction model using artificial intelligence

Journal:	*BMJ Open*
Manuscript ID	bmjopen-2021-052887
Article Type:	Protocol
Date Submitted by the Author:	05-May-2021

Complete List of Authors:	Nadarajah, Ramesh; University of Leeds, Leeds Institute for Data Analytics; University of Leeds, Leeds Institute of Cardiovascular and Metabolic Medicine<br>Wu, Jianhua; University of Leeds, Leeds Institute for Data Analytics; University of Leeds, School of Dentistry<br>Frangi, Alejandro F. ; University of Leeds, School of Computing; University of Leeds, Leeds Institute of Data Analytics<br>Hogg, David; University of Leeds, School of Computing<br>Cowan, Campbell; Leeds Teaching Hospitals NHS Trust, Department of Cardiology<br>Gale, Chris; University of Leeds, Leeds Institute of Cardiovascular and Metabolic Medicine; University of Leeds, Leeds Institute for Data Analytics

Keywords:	PRIMARY CARE, Pacing & electrophysiology < CARDIOLOGY, Adult cardiology < CARDIOLOGY, Cardiac Epidemiology < CARDIOLOGY, Health informatics < BIOTECHNOLOGY & BIOINFORMATICS

SCHOLARONE™ Manuscripts

I, the Submitting Author has the right to grant and does grant on behalf of all authors of the Work (as defined in the below author licence), an exclusive licence and/or a non-exclusive licence for contributions from authors who are: i) UK Crown employees; ii) where BMJ has agreed a CC-BY licence shall apply, and/or iii) in accordance with the terms applicable for US Federal Government officers or employees acting as part of their official duties; on a worldwide, perpetual, irrevocable, royalty-free basis to BMJ Publishing Group Ltd ("BMJ") its licensees and where the relevant Journal is co-owned by BMJ to the co-owners of the Journal, to publish the Work in this journal and any other BMJ products and to exploit all rights, as set out in our licence.

The Submitting Author accepts and understands that any supply made under these terms is made by BMJ to the Submitting Author unless you are acting as an employee on behalf of your employer or a postgraduate student of an affiliated institution which is paying any applicable article publishing charge ("APC") for Open Access articles. Where the Submitting Author wishes to make the Work available on an Open Access basis (and intends to pay the relevant APC), the terms of reuse of such Open Access shall be governed by a Creative Commons licence – details of these licences and which Creative Commons licence will apply to this Work are set out in our licence referred to above.

Other than as permitted in any relevant BMJ Author's Self Archiving Policies, I confirm this Work has not been accepted for publication elsewhere, is not being considered for publication elsewhere and does not duplicate material already published. I confirm all authors consent to publication of this Work and authorise the granting of this licence.

For peer review only

Title: Predicting patient-level new-onset atrial fibrillation from population-based nationwide electronic health records: protocol of FIND-AF for developing a precision medicine prediction model using artificial intelligence

Publication type: Study Protocol

Target journal: BMJ Open

Authors:
Ramesh Nadarajah[1,2,3], Jianhua Wu[2,4], Alejandro F Frangi[1,2,5,6,7], David Hogg[5], Campbell Cowan[3], Chris P Gale[1,2,3]

Affiliations:
[1] Leeds Institute for Cardiovascular and Metabolic Medicine, University of Leeds, UK
[2] Leeds Institute of Data Analytics, University of Leeds, UK
[3] Department of Cardiology, Leeds Teaching Hospitals NHS Trust, Leeds, UK
[4] School of Dentistry, University of Leeds, Leeds, UK
[5] School of Computing, University of Leeds, Leeds, UK
[6] Department of Cardiovascular Sciences, KU Leuven, Leuven, Belgium
[7] Department of Electrical Engineering, KU Leuven, Leuven, Belgium

Correspondence:
Ramesh Nadarajah
British Heart Foundation Clinical Research Fellow
Leeds Institute for Cardiovascular and Metabolic Medicine
University of Leeds
6 Clarendon Way
Leeds, UK
LS2 9DA
Tel +44 (0) 113 343 3241
Email r.nadarajah@leeds.ac.uk
Twitter @Dr_R_Nadarajah

Word Count
3543

Keywords
Atrial Fibrillation
Prediction
Artificial Intelligence
Precision Medicine
Screening
Primary care
Electronic health records

For peer review only

Abstract

Introduction

Atrial fibrillation (AF) is a major cardiovascular health problem: it is common, chronic and incurs substantial healthcare expenditure because of stroke. Oral anticoagulation reduces the risk of thromboembolic stroke in those at higher risk, but for a number of patients stroke is the first manifestation of undetected AF. There is rationale for the early diagnosis of AF, before the first complication occurs, but population-based screening is not recommended. Previous prediction models have been limited by their data sources and methodologies. An accurate model that utilises existing routinely-collected data is needed to inform clinicians of patient-level risk of AF, inform national screening policy and highlight predictors that may be amenable to primary prevention.

Methods and analysis

We will investigate the application of a range of deep learning techniques, including an adapted convolutional neural network (CNN), recurrent neural network (RNN) and Transformer, on routinely-collected primary care data to create a personalised model predicting the risk of new-onset AF over a range of time periods. The Clinical Practice Research Datalink (CPRD)-GOLD dataset will be used for derivation, and the CPRD-AURUM dataset will be used for external geographical validation. Both comprise a sizeable representative population and are linked at patient-level to secondary care databases. The performance of the deep learning models will be compared against classic machine learning and traditional statistical predictive modelling methods. We will only use risk factors accessible in primary care and endow the model with the ability to update risk prediction as it is presented with new data, to make the model more useful in clinical practice.

Ethics and dissemination

Permissions for CPRD-GOLD and CPRD-AURUM datasets were obtained from CPRD (ref no: 19_076). The CPRD ethical approval committee approved the study. The results will be submitted as a research paper for publication to a peer-reviewed journal and presented at peer-reviewed conferences.

Trial registration details

A systematic review to incorporate within the overall project was registered on PROSPERO (registration number CRD42021245093). The study was registered on Clinical Trials.gov (NCT04657900).

For peer review only

Strengths and limitations of the study

- Large and nationwide dataset representative of the United Kingdom (UK) primary care population.
- Using artificial intelligence technology may discover new predictive variables by efficiently incorporating temporal information of event data.
- The model will not just predict the risk of a patient developing AF, but also provide a representation of how risk develops over time to enable more focussed screening.
- The derivation and validation work will be undertaken in datasets in the UK; therefore, further validation work will be needed for international contexts.
- The derivation data will not include unstructured natural language free text; future research could explore if incorporating free text into representation learning improves predictive accuracy

For peer review only

INTRODUCTION

Atrial fibrillation (AF) is the most common sustained cardiac arrhythmia. The current estimated prevalence is between 2% and 4%, and a 2.3-fold rise is expected due to extended longevity in the general population.[1] Whilst AF may present with symptomatic palpitations, for many patients, the first diagnosis of AF is only after they present with stroke or cardiac decompensation. The frequency of AF in ischaemic strokes is 20-30%, and these are usually severe, resulting in permanent disability or fatality.[2]

Oral anticoagulants can reduce the risk of stroke by up to two thirds in those with AF at higher risk of stroke.[3] International guidelines recommend that patients with AF at elevated thromboembolic risk are offered stroke prophylaxis with an oral anticoagulant.[1] Most patients with AF will have stroke risk factors, making them eligible for an oral anticoagulant. Many will have concomitant cardiovascular disease (such as hypertension, valvular heart disease or heart failure) making them eligible for further investigation or treatment. Equally, in those with AF who are low risk for stroke (and therefore do not qualify for oral anticoagulation), surveillance for increasing stroke risk is advisable.

Thus the early diagnosis of AF, before the manifestation of the first complication, remains a major public health challenge. Screening for AF in the community has been proposed as an approach to optimise early AF detection.[4] Previous studies have shown that the risk of atrial fibrillation (often asymptomatic) increases with age.[5] Opportunistic screening is cost-effective in patients aged 65 or over, and amongst 75-76-year old individuals undergoing a 2-week intermittent ECG screening.[6-8] Nevertheless, there is no current recommendation for UK population-wide systematic screening.[9]

Prediction models could contribute to AF screening by discriminating patients into risk categories, from which investigation intensity could be planned.[10] However, models based solely on analysis of investigations may not apply in the community setting - for example, routine ECGs are not always available.[11]

To date, several multivariable prediction models have been created or tested for prediction of incident AF in the community. The earliest models were derived from structured follow-up of prospective cohorts, including FHS-AF (Framingham Heart Study score for Atrial Fibrillation) and CHARGE-AF (Cohorts for Heart and Ageing Research in Genomic Epidemiology for Atrial Fibrillation).[12 13] The proliferation of electronic health records (EHRs) has led to the development of several models from local registries, including MHS (Maccabi Healthcare Services) and C_2HEST (Coronary artery disease/chronic obstructive pulmonary disease, Hypertension, Elderly, Systolic heart failure, Thyroid disease).[14 15] Structured EHRs offer larger sample sizes to assess more candidate variables and derive more generalisable models.

A systematic review found that the models derived from prospective cohorts had been more frequently externally validated. CHARGE-AF was the only model that showed significant overall discrimination in meta-analysis but its transportability to EHRs is still being investigated.[16 17] Each model to date is, however, limited by one or more of their use of small, geographically remote or historical datasets, lack of temporal information, crude risk modelling with consequent suboptimal model performance and/or predictor variables not readily available in primary care.[18] None have yet reached widespread clinical practice.

For peer review only

Machine learning is a data-driven approach that can identify non-linear associations and complex interactions between variables without the need to specify these relationships *a priori*.[19] A recent study applying this methodology to a nationwide-UK dataset produced a model with a greater discriminative capability than CHARGE-AF (Area Under Receiver Operating Characteristic (AUC) 0.827 vs. 0.725) in EHR.[19]

Artificial intelligence (AI) has several desirable features for prediction modelling from EHRs. It facilitates the use of vast quantities of event data and associated temporal information, handles many predictors with automatic variable selection techniques, accommodates non-linearities and interactions among variables, and enables a live learning approach (whereby the prediction model is automatically updated). A range of AI techniques have been applied to EHR data and have demonstrated better prediction power over traditional statistical approaches.[20] Furthermore deep learning, a sub-field of machine learning, can learn complex patterns from data to characterise higher-level correlations among clinical events.[21] Accordingly, it may derive robust patient representations from raw EHR data for prediction modelling without the need for manual, expert-dependent feature engineering like classical machine learning techniques, which places a limit on scalability and generalisability.[22]

Using AI to develop a predictive algorithm from routinely-collected primary care EHRs could offer several advantages:

1) A model could predict the risk that a person will develop new-onset AF, and how that evolves over time, whereas current prediction models only provide a fixed prediction horizon. This would allow phenotype- and temporal-specific screening which could make screening more efficacious and cost-effective.
2) A model created from routinely-collected EHRs could be more smoothly translated into clinical practice by being embedded into existing clinical EHRs.
3) The predictive magnitude of variables for the development of AF may identify novel risk markers for AF, which could then be studied for causality.

For peer review only

Research Aim

The aims of the Future Innovations in Novel Detection of Atrial Fibrillation (FIND-AF) study are to:

1) Develop a deep learning model for predicting the risk, *and evolution of the risk,* of new-onset AF in primary care
2) Identify and quantify the magnitude of risk markers of new AF amongst routinely-collected primary care data
3) Externally validate the model in a geographically distinct dataset to assess generalisability

For peer review only

METHODS AND ANALYSIS

Data sources and permissions

The derivation dataset for training and testing the model will be the Clinical Practice Research Datalink-GOLD (CPRD-GOLD) dataset. This is an ongoing primary care database, established in 1987, that comprises anonymised medical records and prescribing data contributed by general practices using Vision® software. It contains data for approximately 17.5 million patients, with 30% of contributing practices in England, and represents the United Kingdom (UK) population in terms of age, sex and ethnicity.[23] In order to contribute to the database, general practices and other health centres must meet prespecified standards for research-quality data ("up-to-standard").[23 24]

To ascertain whether the prediction model is generalisable, we will evaluate its performance using an external dataset, CPRD-AURUM. This was launched in 2017 and encompasses practices using EMIS Web® software. It contains data for approximately 26.9 million patients and draws on data collected from practices in England only.[25] It is a geographically distinct dataset as any practices which previously contributed to CPRD-GOLD (and now contribute to CPRD-AURUM) have been removed from the CPRD-GOLD cohort. CPRD undertakes various levels of validation and quality assurance.[25]

Recorded information in both datasets includes patients' demography, clinical symptoms, signs, investigations, diagnoses, prescriptions, referrals, behavioural factors and test results entered by clinicians and other practice staff. All clinical information is coded using Read Codes in CPRD-GOLD and SNOMED clinical terms (CT) in CPRD-AURUM.[26 27] In the proposed study, extracted patients will have patient-level data linked to Hospital Episode Statistics (HES) Admitted Patient Care (APC) and Diagnostic Imaging Dataset (DID), Office for National statistics (ONS) Death Registration, patient-level deprivation and practice-level deprivation to provide a more comprehensive dataset. The CPRD dataset has been used to develop or validate a range of risk prediction models, including in cardiovascular disease.[19 28]

The extracted datasets, including linked data, comprise all patients for the period between 2nd January 1998 and 30th November 2018 from the snapshot of CPRD-GOLD and CPRD-AURUM provided in October 2019. Over this study period, the CPRD-GOLD dataset comprises approximately 4.5 million patients eligible for data linkage at an up-to-standard practice, with over 200,000 patients having a record of AF. The CPRD-AURUM dataset comprises approximately 18 million patients eligible for data linkage, with almost 800,000 patients having a record of AF. A sample of 245 general practices will be randomly selected from 800 general practices in CPRD-AURUM to approximately match the size of CPRD-GOLD.

Patient and Public Involvement

Patients and public were not involved in the design of this research. However, a Scientific Advisory Board, including representatives from the Arrhythmia Alliance, National Institute for Health and Care Excellence (NICE) atrial fibrillation guideline committee lay members (last updated 04/01/2020) and EHR software providers, has been created to provide expert context advice on the research, the dissemination of results and advise on the translation of the findings of this study into clinical practice.

For peer review only

Inclusion and Exclusion Criteria

The study population will comprise all available patients in CPRD-GOLD and CPRD-AURUM eligible for data linkage and with at least 1-year follow-up in the period between 2nd January 1998 and 30th November 2018. Patients will be excluded if they were 18 years of age or under at the date of the first registration in CPRD, diagnosed with AF or atrial flutter (AFl) before 1st January 1998, registered for less than 1 year in CPRD or ineligible for data linkage.

Outcome Ascertainment

The outcome of interest is first diagnosed AF or AFl after baseline (1st January 2009). We have included AFl as an outcome since it has similar clinical relevance, including thromboembolic risk and anticoagulation guidelines, as AF.[1] These will be identified using Read codes and SNOMED CT in CPRD datasets. For HES APC events and underlying cause of death variable in the ONS Death Registration data file, the tenth revision of the International Statistical Classification of Diseases and Related Health Problems (ICD-10) codes will be used.

Sample size

To develop a prognostic prediction model, the required sample size may be determined by three criteria suggested by *Riley et al*.[29] For example, suppose a maximum of 200 parameters will be included in the prediction model and the Cox-Snell generalised R^2 is assumed to be 0.01. A total of 377,996 patients will be required to meet Riley's criterion (i) with global shrinkage factor of 0.95; this sample size also ensures a small absolute difference ($\Delta < 0.05$) in the apparent and adjusted Nagelkerke R^2 (Riley's criterion (ii)) and ensures precise estimate of overall risk with a margin of error < 0.001 (Riley's criterion (iii)). According to the Quality and Outcomes Framework (QOF), the prevalence of AF in England is 1.7%.[30 31] Given an AF prevalence of 1.7%, only 6,425 patients will be expected to develop AF from 377,996 patients. Therefore, the number of patients in the CPRD dataset with AF will provide sufficient statistical power to develop and validate a deep learning prediction algorithm with the predefined precision and accuracy.

Predictor Variables

A systematic review has highlighted 22 predictor variables included in varying combinations by 10 preceding prediction models developed to detect incident AF in the community.[16] In addition, a more recently-published machine learning model, has established six further time-varying variables (e.g. change in body mass index between the latest two quarters).[19]

To capture the full potential of deep learning and this large dataset, we will broaden our search for candidate predictors to all available variables, whilst retaining temporal information (including all clinical assessments, hospitalised events and medications). The potential predictors may include:

For peer review only

1. Sociodemographic variables including age, sex, ethnicity and indices of multiple deprivation.
2. All disease conditions during follow-up, including hospitalised diseases and procedures, such as other cardiovascular diseases, diabetes mellitus, chronic lung disease, renal disease, inflammatory disease, cancer, hypothyroidism and surgical procedures.
3. Clinical assessments including heart rate, systolic and diastolic blood pressure, height, weight and body mass index.
4. Medications prescribed including antihypertensives, statins, antidepressants, anxiolytics/hypnotics and antipsychotics.
5. Lifestyle factors including smoking status and alcohol consumption.
6. All biomarkers routinely collected during follow-up including total, high-density lipoprotein and low-density lipoprotein cholesterol, triglycerides, creatinine, c-reactive protein, erythrocyte sedimentation rate.

Predictive factors will be identified using the appropriate codes. In CPRD, we will use Read codes for diagnoses, measurements (e.g. systolic and diastolic values) and product codes (prodcodes) for medications. In HES APC, we will use ICD-10 codes and Office of Population Censuses and Surveys Classification of Interventions and Procedures version 4 (OPCS-4) codes. In the ONS Death Registration data file, we will use ICD-10 codes (and ICD-9 codes for the period before 2001).

<u>Missing data</u>

Missing data is expected in EHR data, and will be handled using multiple imputations according to the approaches suggested by Carpenter and Kenward, and depending on the amount of missing data.[32] It is likely there will be misclassification of baseline characteristics, such as smoking status, cholesterol, blood pressure and weight that change over time. To account for this, we will define baseline information using only measures recorded within the last year. We will then use measures taken within the last 2-5 years and in the year after baseline as parameters in the chained imputation equations used to impute baseline covariates with $\leq$ 40% missingness. In addition, median imputation, a common approach to deal with missing values in machine learning algorithms will be used to test the model robustness.[33]

<u>Data analysis plan</u>

Development and external validation of model

The CPRD-GOLD and CPRD-AURUM data will be cleaned and preprocessed for model development and validation, respectively. Specifically, for patient features with binary values, 0 and 1 will be mapped to the binary values. Variables with multiple categories will be split into their component categories, and each given a binary value to indicate the presence or not of the variable for each patient. Continuous variables will be kept as continuous. To reduce the high cardinality of Read codes and ICD codes we will map both to Caliber codes,[34] which is an expert checked mapping directory from University College London. Prodcodes can be mapped to level 2 of BNF (British National Formulary) codes.[35]

For peer review only

A number of deep learning models will be investigated for prediction of AF in CPRD-GOLD. These will include a convolutional neural network (CNN), a recurrent neural network (RNN) and a Transformer architecture. They each possess characteristics that can capture the temporality of EHR data and the progression of a patient's health status.

Although CNNs are typically associated with static content (e.g. images and documents) they are increasingly used to uncover temporal relationships in EHR.[36-39] The patient EHR can be converted to a temporal matrix, as proposed in *Cheng et al.* with one dimension corresponding to time and the other dimension corresponding to medical events (Figure 1a).[36] A one-sided convolution operation can be applied to each possible window of features in the event matrix to produce a feature map followed with max-pooling (to capture the most important features) and culminating in a fully connected layer and softmax classifier. If the time dimension is embedded in 1 day increments, we have the option to learn temporal features by extending connectivity in the time dimension through a range of fusion strategies. Temporal early fusion could combine information across an entire time window to establish global patterns, temporal late fusion can be performed in the fully connected layer to model local connections, and temporal slow fusion could capture both by extending the connectivity of all convolutional layers.[36]

Patient EHR events including diagnoses, procedures and medications can also be represented as a sequence of codes over time, similar to words in a sentence (Figure 1b). After embedding into a lower dimensional space a one-dimensional convolutional operation over the temporal dimension with a combination of filters of different lengths could capture temporal dependencies at multiple levels as proposed in *Che et al*.[37]

A sequence-based representation also makes patient EHR amenable to techniques that have provided breakthroughs in natural language processing, especially RNNs.[40-42] Recurrent neural networks with a gated recurrent unit (GRU) model design have been successfully applied in modelling sequential structured EHR data to predict diagnoses as they can accept an input vector at each time step while storing information in a hidden layer which changes over time.[21 43-50] We will apply the reverse time attention model (RETAIN), which has been tested for the prediction of heart failure in CPRD.[51] RETAIN can generate temporal attention vectors at both visit and variable-level by running RNNs backwards, which somewhat mimics a physician's behaviours in examining a patient's past visits in reverse time order and could capture the short-term time-varying events that were found to improve prediction in a previous shallow neural network model.[19 45] Timestamps can be included to calculate the attentions for the entire visit sequence and thus provide temporal information for each visit.[45]

Finally, Transformers have become state-of-art in natural language processing,[52 53] and we will apply an adaption for EHR, BEHRT (BERT for EHR), which was originally developed in CPRD.[54] This model's structure, depicting each diagnosis code as a word, with each visit as a sentence and the entire medical history as a document facilitates multi-head self-attention, positional encoding and masked language model for EHR. BEHRT can define a representation incorporating information on the diseases afflicting a patient, the positional interactions of diseases and sequence of events, with the age of a patient linked to each visit to give a sense of time between diagnoses.[54] This representation can then be used for a number of tasks including disease prediction.

Preprocessed patient-level data in CPRD-GOLD will be randomly split into an 80:20 ratio to create training and testing samples. The split ratio is not a significant factor, given the volume of the sample size. The model parameters and dropout rate, will be chosen through a grid search and 10-fold cross-validation will be used (i.e. 10% of the training data will be randomly selected as the cross-validation set).

Whilst the outcome is binary, instead of just predicting 0 or 1 for a patient (new AF or not), the probability of that patient developing AF by different time points over months (1-, 3-, 6-) and years (1-, 5-, 10-) msy be predicted. The probability of developing AF at each time point could be plotted to give an understanding of evolving risk of AF. The clinical risk prediction performance of the deep learning models will be compared against a range of classic machine learning techniques and traditional statistical predictive modelling methods including support vector machine (SVM), random forest (RF), naïve Bayes (NB) and Cox proportional hazards model (CPH). Discrimination (Harrell's c-statistic and area under receiver operating characteristics) and calibration metrics will be supplemented with positive and negative predictive values, precision, recall and area under precision-recall curve for all models.

The CPRD-AURUM dataset will then externally validate the model performance to assess generalisability. It has been shown that a lack of external validation has hampered the implementation of previous machine learning models in routine clinical practice.[55]

Identification and quantification of the magnitude of predictors

The proposed deep learning models can extract informative risk factors from EHR data. Specifically, a risk factor selection strategy proposed in *Huang et al.* will be adapted to identify informative risk factors.[56] The model will provide weights of the identified risk factors to help understand the significance of risk factors at different risk levels. The impact of the number of risk factors on AF risk prediction performance will be assessed through the curves of both AUC and prediction accuracy plotted against the number of risk factors. Some predictors, such as body mass index, blood pressure, frequency of visits and strength of the prescribed medication, may change over time. The incremental prognostic values of including these variable trajectories will be explored, and the impact on predictive accuracy will be assessed.

<u>Software</u>

The deep learning model will be implemented in R (through the R studio interface) and TensorFlow or Python and PyTorch including data preprocessing, missing data imputation, model development and validation.

For peer review only - http://bmjopen.bmj.com/site/about/guidelines.xhtml

ETHICS AND DISSEMINATION

The study has been approved by CPRD (ref no: 19_076). Those handling data have completed University of Leeds information security training. All analyses will be conducted in concordance with the CPRD study dataset agreement between the Secretary of State for Health and Social Care and the University of Leeds. The study has been registered at clinical trials.gov (NCT04657900).

The study is informed by the Prognosis Research Strategy (PROGRESS) framework and recommendations.[57] The subsequent research paper will be submitted for publication in a peer-reviewed journal and will be written following TRIPOD: *transparent reporting of a multivariable prediction model for individual prognosis or diagnosis* and RECORD: *reporting of studies conducted using observational routinely-collected health data* guidelines.[58 59]

If the model succeeds (defined as improving predicting accuracy by at least 5% compared with existing models), the algorithm could be made readily available through free-to-use software. The model will be designed to be amenable to in-situ updating with new information so that prediction of an individual's AF risk is updated contemporaneously. The algorithm could be a built-in tool for use in general practices to 'screen' for patients at high risk of developing new onset AF. Future research will be needed to assess the clinical impact of this risk model. At the point when utilisation in clinical practice is possible the applicable regulation on medicine devices will be adhered to.[60] When in clinical use, the model itself could also be reviewed and updated by a pre-specified expert consensus group on an annual basis after incorporating evidence from post-service utilization and the curation of more data.

For peer review only

CONCLUSIONS

Atrial Fibrillation is a common clinical problem with potentially catastrophic sequelae. A prediction model that may identify in a community setting which individuals will develop AF, and when this is most likely to occur, could enable targeted screening. This British Heart Foundation funded study will contribute to knowledge about the detection of AF through prediction using a data science approach in routine EHR data. The use of artificial intelligence technology may uncover new predictors in EHR and facilitate easier translation into clinical practice.

For peer review only

Authors' contributions
JW and CPG conceived the concept and JW planned the analysis. RN wrote the first draft, with contributions from all authors. All authors (RN, JW, DH, AF, CC, CPG) approved the final version and jointly take responsibility for the decision to submit the manuscript to be considered for publication.

Funding statement
RN is supported by the British Heart Foundation Clinical Research Training Fellowship (FS/20/12/34789).
The Clinical Practice Research Datalink (CPRD) is a joint venture from the Medicines and Healthcare Regulatory Agency (MHRA) and the National Institute for Health Research (NIHR). The CPRD is owned by the United Kingdom (UK) Department of Health.

Competing Interests
None declared

For peer review only

REFERENCES

1. Hindricks G, Potpara T, Dagres N, et al. 2020 ESC Guidelines for the diagnosis and management of atrial fibrillation developed in collaboration with the European Association of Cardio-Thoracic Surgery (EACTS). *Eur Heart J* 2020 doi: 10.1093/eurheartj/ehaa612 [published Online First: 2020/08/30]
2. Benjamin EJ, Virani SS, Callaway CW, et al. American heart association council on epidemiology and prevention statistics committee and stroke statistics subcommittee. *Heart disease and stroke statistics-2018 update: a report from the American Heart Association Circulation* 2018;137(12):e67-e492.
3. Ruff CT, Giugliano RP, Braunwald E, et al. Comparison of the efficacy and safety of new oral anticoagulants with warfarin in patients with atrial fibrillation: a meta-analysis of randomised trials. *Lancet* 2014;383(9921):955-62. doi: 10.1016/S0140-6736(13)62343-0 [published Online First: 2013/12/10]
4. Freedman B, Camm J, Calkins H, et al. Screening for atrial fibrillation: a report of the AF-SCREEN International Collaboration. *Circulation* 2017;135(19):1851-67.
5. Boriani G, Laroche C, Diemberger I, et al. Asymptomatic atrial fibrillation: clinical correlates, management, and outcomes in the EORP-AF Pilot General Registry. *Am J Med* 2015;128(5):509-18 e2. doi: 10.1016/j.amjmed.2014.11.026 [published Online First: 2014/12/24]
6. Aronsson M, Svennberg E, Rosenqvist M, et al. Cost-effectiveness of mass screening for untreated atrial fibrillation using intermittent ECG recording. *Ep Europace* 2015;17(7):1023-29.
7. Hobbs FR, Fitzmaurice D, Mant J, et al. A randomised controlled trial and cost-effectiveness study of systematic screening (targeted and total population screening) versus routine practice for the detection of atrial fibrillation in people aged 65 and over. The SAFE study. 2005
8. Svennberg E, Engdahl J, Al-Khalili F, et al. Mass screening for untreated atrial fibrillation: the STROKESTOP study. 2015;131(25):2176-84.
9. Committee UNS. The UK NSC recommendation on Atrial Fibrillation screening in adults 2019 [cited 2021 April 26]. Available from: https://legacyscreening.phe.org.uk/atrialfibrillation#:~:text=The%20UK%20NSC%20does%20not,in%20people%20found%20through%20screening.
10. Moons KG, Kengne AP, Woodward M, et al. Risk prediction models: I. Development, internal validation, and assessing the incremental value of a new (bio) marker. *Heart* 2012;98(9):683-90.
11. Attia ZI, Noseworthy PA, Lopez-Jimenez F, et al. An artificial intelligence-enabled ECG algorithm for the identification of patients with atrial fibrillation during sinus rhythm: a retrospective analysis of outcome prediction. *The Lancet* 2019;394(10201):861-67.
12. Alonso A, Krijthe BP, Aspelund T, et al. Simple risk model predicts incidence of atrial fibrillation in a racially and geographically diverse population: the CHARGE-AF consortium. *J Am Heart Assoc* 2013;2(2):e000102. doi: 10.1161/JAHA.112.000102 [published Online First: 2013/03/30]
13. Schnabel RB, Sullivan LM, Levy D, et al. Development of a risk score for atrial fibrillation (Framingham Heart Study): a community-based cohort study. *The Lancet* 2009;373(9665):739-45. doi: 10.1016/s0140-6736(09)60443-8

For peer review only - http://bmjopen.bmj.com/site/about/guidelines.xhtml

14. Aronson D, Shalev V, Katz R, et al. Risk Score for Prediction of 10-Year Atrial Fibrillation: A Community-Based Study. *Thromb Haemost* 2018;118(9):1556-63. doi: 10.1055/s-0038-1668522 [published Online First: 2018/08/14]
15. Li YG, Pastori D, Farcomeni A, et al. A Simple Clinical Risk Score (C2HEST) for Predicting Incident Atrial Fibrillation in Asian Subjects: Derivation in 471,446 Chinese Subjects, With Internal Validation and External Application in 451,199 Korean Subjects. *Chest* 2019;155(3):510-18. doi: 10.1016/j.chest.2018.09.011 [published Online First: 2018/10/08]
16. Himmelreich JC, Veelers L, Lucassen WA, et al. Prediction models for atrial fibrillation applicable in the community: a systematic review and meta-analysis. *EP Europace* 2020;22(5):684-94.
17. Himmelreich JC, Lucassen WA, Harskamp RE, et al. CHARGE-AF in a national routine primary care electronic health records database in the Netherlands: validation for 5-year risk of atrial fibrillation and implications for patient selection in atrial fibrillation screening. 2021;8(1):e001459.
18. Kolek MJ, Graves AJ, Xu M, et al. Evaluation of a prediction model for the development of atrial fibrillation in a repository of electronic medical records. *JAMA cardiology* 2016;1(9):1007-13.
19. Hill NR, Ayoubkhani D, McEwan P, et al. Predicting atrial fibrillation in primary care using machine learning. *PloS one* 2019;14(11):e0224582.
20. Weng SF, Reps J, Kai J, et al. Can machine-learning improve cardiovascular risk prediction using routine clinical data? *PloS one* 2017;12(4):e0174944.
21. Health-atm: A deep architecture for multifaceted patient health record representation and risk prediction. Proceedings of the 2018 SIAM International Conference on Data Mining; 2018. SIAM.
22. Chen D, Liu S, Kingsbury P, et al. Deep learning and alternative learning strategies for retrospective real-world clinical data. 2019;2(1):1-5.
23. Herrett E, Gallagher AM, Bhaskaran K, et al. Data resource profile: clinical practice research datalink (CPRD). *International journal of epidemiology* 2015;44(3):827-36.
24. Herrett E, Thomas SL, Schoonen WM, et al. Validation and validity of diagnoses in the General Practice Research Database: a systematic review. *British journal of clinical pharmacology* 2010;69(1):4-14.
25. Wolf A, Dedman D, Campbell J, et al. Data resource profile: Clinical practice research datalink (cprd) aurum. *International journal of epidemiology* 2019;48(6):1740-40g.
26. Chisholm J. The Read clinical classification. *BMJ: British Medical Journal* 1990;300(6732):1092.
27. SNOMED clinical terms: overview of the development process and project status. Proceedings of the AMIA Symposium; 2001. American Medical Informatics Association.
28. Hippisley-Cox J, Coupland C, Brindle P. Development and validation of QRISK3 risk prediction algorithms to estimate future risk of cardiovascular disease: prospective cohort study. *bmj* 2017;357:j2099.
29. Riley RD, Snell KI, Ensor J, et al. Minimum sample size for developing a multivariable prediction model: PART II-binary and time-to-event outcomes. *Statistics in medicine* 2019;38(7):1276-96.

For peer review only

30. Cowan JC, Wu J, Hall M, et al. A 10 year study of hospitalized atrial fibrillation-related stroke in England and its association with uptake of oral anticoagulation. *European heart journal* 2018;39(32):2975-83.
31. Wu J, Alsaeed ES, Barrett J, et al. Prescription of oral anticoagulants and antiplatelets for stroke prophylaxis in atrial fibrillation: nationwide time series ecological analysis. *EP Europace* 2020;22(9):1311-19.
32. Carpenter J, Kenward M. Multiple imputation and its application: John Wiley & Sons 2012.
33. Batista GE, Monard MC. An analysis of four missing data treatment methods for supervised learning. *Applied artificial intelligence* 2003;17(5-6):519-33.
34. Kuan V, Denaxas S, Gonzalez-Izquierdo A, et al. A chronological map of 308 physical and mental health conditions from 4 million individuals in the English National Health Service. 2019;1(2):e63-e77.
35. BNF Publications [22 April 2021]. Available from: https://www.bnf.org/.
36. Risk prediction with electronic health records: A deep learning approach. Proceedings of the 2016 SIAM International Conference on Data Mining; 2016. SIAM.
37. Che Z, Cheng Y, Sun Z, et al. Exploiting convolutional neural network for risk prediction with medical feature embedding. 2017
38. Development of Deep Learning Algorithm for Detection of Colorectal Cancer in EHR Data. MedInfo; 2019.
39. Suo Q, Ma F, Yuan Y, et al. Deep patient similarity learning for personalized healthcare. 2018;17(3):219-27.
40. Mikolov T, Sutskever I, Chen K, et al. Distributed representations of words and phrases and their compositionality. 2013
41. Zaremba W, Sutskever I, Vinyals OJapa. Recurrent neural network regularization. 2014
42. Shickel B, Tighe PJ, Bihorac A, et al. Deep EHR: a survey of recent advances in deep learning techniques for electronic health record (EHR) analysis. 2017;22(5):1589-604.
43. Doctor ai: Predicting clinical events via recurrent neural networks. Machine learning for healthcare conference; 2016. PMLR.
44. Choi E, Schuetz A, Stewart WF, et al. Using recurrent neural network models for early detection of heart failure onset. 2017;24(2):361-70.
45. Choi E, Bahadori MT, Kulas JA, et al. Retain: An interpretable predictive model for healthcare using reverse time attention mechanism. 2016
46. GRAM: graph-based attention model for healthcare representation learning. Proceedings of the 23rd ACM SIGKDD international conference on knowledge discovery and data mining; 2017.
47. Dipole: Diagnosis prediction in healthcare via attention-based bidirectional recurrent neural networks. Proceedings of the 23rd ACM SIGKDD international conference on knowledge discovery and data mining; 2017.
48. Kwon BC, Choi M-J, Kim JT, et al. Retainvis: Visual analytics with interpretable and interactive recurrent neural networks on electronic medical records. 2018;25(1):299-309.
49. Kame: Knowledge-based attention model for diagnosis prediction in healthcare. Proceedings of the 27th ACM International Conference on Information and Knowledge Management; 2018.
50. Choi E, Xiao C, Stewart WF, et al. Mime: Multilevel medical embedding of electronic health records for predictive healthcare. 2018

For peer review only

51. Solares JRA, Raimondi FED, Zhu Y, et al. Deep learning for electronic health records: A comparative review of multiple deep neural architectures. 2020;101:103337.
52. Devlin J, Chang M-W, Lee K, et al. Bert: Pre-training of deep bidirectional transformers for language understanding. 2018
53. Vaswani A, Shazeer N, Parmar N, et al. Attention is all you need. 2017
54. Li Y, Rao S, Solares JRA, et al. BEHRT: transformer for electronic health records. 2020;10(1):1-12.
55. Banerjee A, Chen S, Fatemifar G, et al. Machine learning for subtype definition and risk prediction in heart failure, acute coronary syndromes and atrial fibrillation: systematic review of validity and clinical utility. 2021;19(1):1-14.
56. Huang Z, Dong W, Duan H, et al. A regularized deep learning approach for clinical risk prediction of acute coronary syndrome using electronic health records. *IEEE Transactions on Biomedical Engineering* 2017;65(5):956-68.
57. Steyerberg EW, Moons KG, van der Windt DA, et al. Prognosis Research Strategy (PROGRESS) 3: prognostic model research. *PLoS Med* 2013;10(2):e1001381.
58. Collins GS, Reitsma JB, Altman DG, et al. Transparent Reporting of a Multivariable Prediction Model for Individual Prognosis or Diagnosis (TRIPOD) The TRIPOD Statement. *Circulation* 2015;131(2):211-19.
59. Nicholls SG, Quach P, von Elm E, et al. The reporting of studies conducted using observational routinely-collected health data (RECORD) statement: methods for arriving at consensus and developing reporting guidelines. *PLoS One* 2015;10(5):e0125620.
60. Kramer DB, Xu S, Kesselheim AS. Regulation of medical devices in the United States and European Union. The Ethical Challenges of Emerging Medical Technologies: Taylor and Francis 2020:41-49.

For peer review only

Patient consent for publication
Not required

Ethics Approval
Permissions for the CPRD-GOLD and CPRD-AURUM datasets were obtained from CPRD (ref no: 19_076). The study was approved by CPRD ethical approval committee.

Word Count
3543

For peer review only

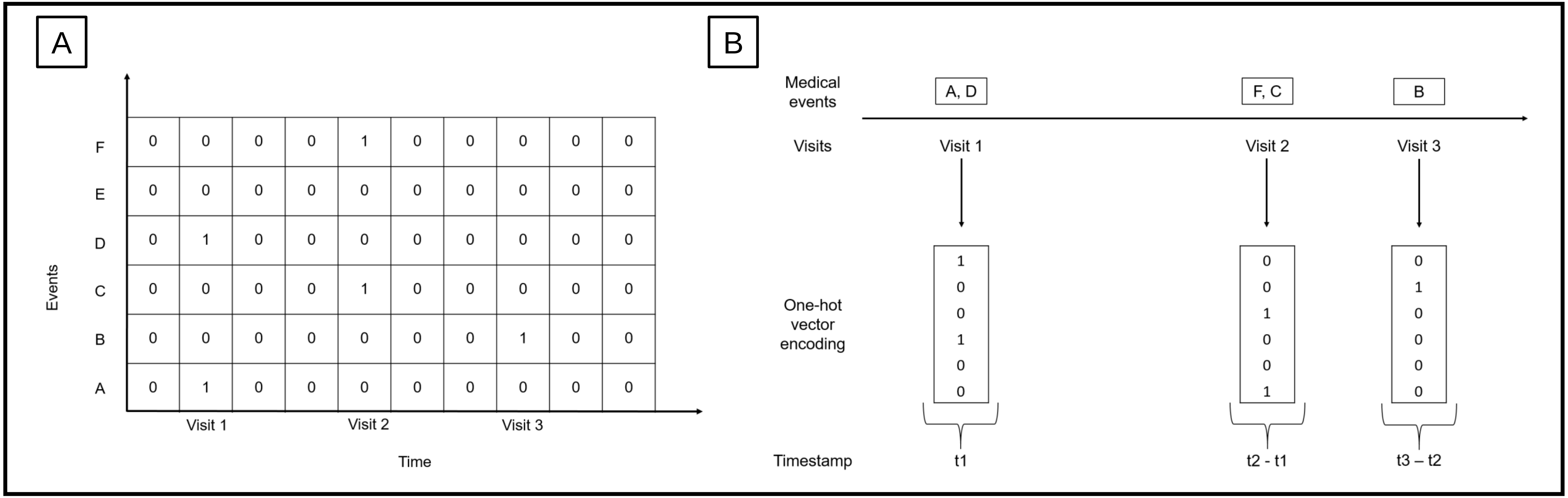

Figure 1 An example of how a patient's EHR could be represented as a temporal matrix (A) compared to a sequence (B). In (A) time is on the x dimension and binary medical events are on the y dimension. In (B) the temporal information, in this example, is represented as inter-visit interval through timestamps (e.g. t2 – t1)

BMJ Open

Predicting patient-level new-onset atrial fibrillation from population-based nationwide electronic health records: protocol of FIND-AF for developing a precision medicine prediction model using artificial intelligence

Journal:	*BMJ Open*
Manuscript ID	bmjopen-2021-052887.R1
Article Type:	Protocol
Date Submitted by the Author:	22-Aug-2021
Complete List of Authors:	Nadarajah, Ramesh; University of Leeds, Leeds Institute for Data Analytics; University of Leeds, Leeds Institute of Cardiovascular and Metabolic Medicine<br>Wu, Jianhua; University of Leeds, Leeds Institute for Data Analytics; University of Leeds, School of Dentistry<br>Frangi, Alejandro F. ; University of Leeds, School of Computing; University of Leeds, Leeds Institute of Data Analytics<br>Hogg, David; University of Leeds, School of Computing<br>Cowan, Campbell; Leeds Teaching Hospitals NHS Trust, Department of Cardiology<br>Gale, Chris; University of Leeds, Leeds Institute of Cardiovascular and Metabolic Medicine; University of Leeds, Leeds Institute for Data Analytics
**Primary Subject Heading**:	Cardiovascular medicine
Secondary Subject Heading:	Epidemiology, General practice / Family practice, Health informatics
Keywords:	PRIMARY CARE, Pacing & electrophysiology < CARDIOLOGY, Adult cardiology < CARDIOLOGY, Cardiac Epidemiology < CARDIOLOGY, Health informatics < BIOTECHNOLOGY & BIOINFORMATICS

SCHOLARONE™
Manuscripts

I, the Submitting Author has the right to grant and does grant on behalf of all authors of the Work (as defined in the below author licence), an exclusive licence and/or a non-exclusive licence for contributions from authors who are: i) UK Crown employees; ii) where BMJ has agreed a CC-BY licence shall apply, and/or iii) in accordance with the terms applicable for US Federal Government officers or employees acting as part of their official duties; on a worldwide, perpetual, irrevocable, royalty-free basis to BMJ Publishing Group Ltd ("BMJ") its licensees and where the relevant Journal is co-owned by BMJ to the co-owners of the Journal, to publish the Work in this journal and any other BMJ products and to exploit all rights, as set out in our licence.

The Submitting Author accepts and understands that any supply made under these terms is made by BMJ to the Submitting Author unless you are acting as an employee on behalf of your employer or a postgraduate student of an affiliated institution which is paying any applicable article publishing charge ("APC") for Open Access articles. Where the Submitting Author wishes to make the Work available on an Open Access basis (and intends to pay the relevant APC), the terms of reuse of such Open Access shall be governed by a Creative Commons licence – details of these licences and which Creative Commons licence will apply to this Work are set out in our licence referred to above.

Other than as permitted in any relevant BMJ Author's Self Archiving Policies, I confirm this Work has not been accepted for publication elsewhere, is not being considered for publication elsewhere and does not duplicate material already published. I confirm all authors consent to publication of this Work and authorise the granting of this licence.

For peer review only

Title: Predicting patient-level new-onset atrial fibrillation from population-based nationwide electronic health records: protocol of FIND-AF for developing a precision medicine prediction model using artificial intelligence

Publication type: Study Protocol

Target journal: BMJ Open

Authors:
Ramesh Nadarajah[1,2,3], Jianhua Wu[2,4], Alejandro F Frangi[1,2,5,6,7], David Hogg[5], Campbell Cowan[3], Chris P Gale[1,2,3]

Affiliations:
[1] Leeds Institute for Cardiovascular and Metabolic Medicine, University of Leeds, UK
[2] Leeds Institute of Data Analytics, University of Leeds, UK
[3] Department of Cardiology, Leeds Teaching Hospitals NHS Trust, Leeds, UK
[4] School of Dentistry, University of Leeds, Leeds, UK
[5] School of Computing, University of Leeds, Leeds, UK
[6] Department of Cardiovascular Sciences, KU Leuven, Leuven, Belgium
[7] Department of Electrical Engineering, KU Leuven, Leuven, Belgium

Correspondence:
Ramesh Nadarajah
British Heart Foundation Clinical Research Fellow
Leeds Institute for Cardiovascular and Metabolic Medicine
University of Leeds
6 Clarendon Way
Leeds, UK
LS2 9DA
Tel +44 (0) 113 343 3241
Email r.nadarajah@leeds.ac.uk
Twitter @Dr_R_Nadarajah

Word Count
3603

Keywords
Atrial Fibrillation
Prediction
Artificial Intelligence
Precision Medicine
Screening
Primary care
Electronic health records

For peer review only

Abstract

Introduction

Atrial fibrillation (AF) is a major cardiovascular health problem: it is common, chronic and incurs substantial healthcare expenditure because of stroke. Oral anticoagulation reduces the risk of thromboembolic stroke in those at higher risk, but for a number of patients stroke is the first manifestation of undetected AF. There is rationale for the early diagnosis of AF, before the first complication occurs, but population-based screening is not recommended. Previous prediction models have been limited by their data sources and methodologies. An accurate model that utilises existing routinely-collected data is needed to inform clinicians of patient-level risk of AF, inform national screening policy and highlight predictors that may be amenable to primary prevention.

Methods and analysis

We will investigate the application of a range of deep learning techniques, including an adapted convolutional neural network (CNN), recurrent neural network (RNN) and Transformer, on routinely-collected primary care data to create a personalised model predicting the risk of new-onset AF over a range of time periods. The Clinical Practice Research Datalink (CPRD)-GOLD dataset will be used for derivation, and the CPRD-AURUM dataset will be used for external geographical validation. Both comprise a sizeable representative population and are linked at patient-level to secondary care databases. The performance of the deep learning models will be compared against classic machine learning and traditional statistical predictive modelling methods. We will only use risk factors accessible in primary care and endow the model with the ability to update risk prediction as it is presented with new data, to make the model more useful in clinical practice.

Ethics and dissemination

Permissions for CPRD-GOLD and CPRD-AURUM datasets were obtained from CPRD (ref no: 19_076). The CPRD ethical approval committee approved the study. The results will be submitted as a research paper for publication to a peer-reviewed journal and presented at peer-reviewed conferences.

Trial registration details

A systematic review to incorporate within the overall project was registered on PROSPERO (registration number CRD42021245093). The study was registered on Clinical Trials.gov (NCT04657900).

For peer review only

Strengths and limitations of the study

- Large and nationwide dataset representative of the United Kingdom (UK) primary care population.
- Using artificial intelligence technology may discover new predictive variables by efficiently incorporating temporal information of event data.
- The model will not just predict the risk of a patient developing AF, but also provide a representation of how risk develops over time to enable more focussed screening.
- The derivation and validation work will be undertaken in datasets collected over the same time period in the UK; therefore, further validation work may be pursued with newly-collected data and for international contexts.
- The derivation data will not include unstructured natural language free text; future research could explore if incorporating free text into representation learning improves predictive accuracy

For peer review only

INTRODUCTION

Atrial fibrillation (AF) is the most common sustained cardiac arrhythmia. The current estimated prevalence is between 2% and 4%, and a 2.3-fold rise is expected due to extended longevity in the general population.[1] Whilst AF may present with symptomatic palpitations, for many patients, the first diagnosis of AF is only after they present with stroke or cardiac decompensation. The frequency of AF in ischaemic strokes is 20-30%, and these are usually severe, resulting in permanent disability or fatality.[2]

Oral anticoagulants can reduce the risk of stroke by up to two thirds in those with AF at higher risk of stroke.[3] International guidelines recommend that patients with AF at elevated thromboembolic risk are offered stroke prophylaxis with an oral anticoagulant.[1] Most patients with AF will have stroke risk factors, making them eligible for an oral anticoagulant. Many will have concomitant cardiovascular disease (such as hypertension, valvular heart disease or heart failure) making them eligible for further investigation or treatment. Equally, in those with AF who are low risk for stroke (and therefore do not qualify for oral anticoagulation), surveillance for increasing stroke risk is advisable.

Thus the early diagnosis of AF, before the manifestation of the first complication, remains a major public health challenge. Screening for AF in the community has been proposed as an approach to optimise early AF detection.[4] Previous studies have shown that the risk of atrial fibrillation (often asymptomatic) increases with age.[5] Opportunistic screening is cost-effective in patients aged 65 or over, and amongst 75-76-year old individuals undergoing a 2-week intermittent ECG screening.[6-8] Nevertheless, there is no current recommendation for UK population-wide systematic screening.[9]

Prediction models could contribute to AF screening by discriminating patients into risk categories, from which investigation intensity could be planned.[10] However, models based solely on analysis of investigations may not apply in the community setting - for example, routine ECGs are not always available.[11]

To date, several multivariable prediction models have been created or tested for prediction of incident AF in the community. The earliest models were derived from structured follow-up of prospective cohorts, including FHS-AF (Framingham Heart Study score for Atrial Fibrillation) and CHARGE-AF (Cohorts for Heart and Ageing Research in Genomic Epidemiology for Atrial Fibrillation).[12 13] The proliferation of electronic health records (EHRs) has led to the development of several models from local registries, including MHS (Maccabi Healthcare Services) and C_2HEST (Coronary artery disease/chronic obstructive pulmonary disease, Hypertension, Elderly, Systolic heart failure, Thyroid disease).[14 15] Structured EHRs offer larger sample sizes to assess more candidate variables and derive more generalisable models.

A systematic review found that the models derived from prospective cohorts had been more frequently externally validated. CHARGE-AF was the only model that showed significant overall discrimination in meta-analysis but its transportability to EHRs is still being investigated.[16 17] Each model to date is, however, limited by one or more of their use of small, geographically remote or historical datasets, lack of temporal information, crude risk modelling with consequent suboptimal model performance and/or predictor variables not readily available in primary care.[18] None have yet reached widespread clinical practice.

For peer review only

Machine learning is a data-driven approach that can identify non-linear associations and complex interactions between variables without the need to specify these relationships *a priori*.[19] A recent study applying this methodology to a nationwide-UK dataset produced a model with a greater discriminative capability than CHARGE-AF (Area Under Receiver Operating Characteristic (AUC) 0.827 vs. 0.725) in EHR.[19]

Artificial intelligence (AI) has several desirable features for prediction modelling from EHRs. It facilitates the use of vast quantities of event data and associated temporal information, handles many predictors with automatic variable selection techniques, accommodates non-linearities and interactions among variables, and enables a live learning approach (whereby the prediction model is automatically updated). A range of AI techniques have been applied to EHR data and have demonstrated better prediction power over traditional statistical approaches.[20] Furthermore deep learning, a sub-field of machine learning, can learn complex patterns from data to characterise higher-level correlations among clinical events.[21] Accordingly, it may derive robust patient representations from raw EHR data for prediction modelling without the need for manual, expert-dependent feature engineering like classical machine learning techniques, which places a limit on scalability and generalisability.[22]

Using AI to develop a predictive algorithm from routinely-collected primary care EHRs could offer several advantages:

1) A model could predict the risk that a person will develop new-onset AF, and how that evolves over time, whereas current prediction models only provide a fixed prediction horizon. This would allow phenotype- and temporal-specific screening which could make screening more efficacious and cost-effective.
2) A model created from routinely-collected EHRs could be more smoothly translated into clinical practice by being embedded into existing clinical EHRs.
3) The predictive magnitude of variables for the development of AF may identify novel risk markers for AF, which could then be studied for causality.

For peer review only

Research Aim

The aims of the Future Innovations in Novel Detection of Atrial Fibrillation (FIND-AF) study are to:

1) Develop a deep learning model for predicting the risk, *and evolution of the risk,* of new-onset AF in primary care
2) Identify and quantify the magnitude of risk markers of new AF amongst routinely-collected primary care data
3) Externally validate the model in a geographically distinct dataset to assess generalisability

For peer review only

METHODS AND ANALYSIS

Data sources and permissions

The derivation dataset for training and testing the model will be the Clinical Practice Research Datalink-GOLD (CPRD-GOLD) dataset. This is an ongoing primary care database, established in 1987, that comprises anonymised medical records and prescribing data contributed by general practices using Vision® software. It contains data for approximately 17.5 million patients, with 30% of contributing practices in England, and represents the United Kingdom (UK) population in terms of age, sex and ethnicity.[23] In order to contribute to the database, general practices and other health centres must meet prespecified standards for research-quality data ("up-to-standard").[23 24]

To ascertain whether the prediction model is generalisable, we will externally validate its performance in the geographically distinct CPRD-AURUM dataset. This was launched in 2017 and encompasses only practices using EMIS Web® software. It contains data for approximately 26.9 million patients and draws on data collected from practices in England only.[25] Any practices which previously contributed to CPRD-GOLD have been removed from the CPRD-GOLD cohort to ensure that these datasets reflect different populations. CPRD undertakes various levels of validation and quality assurance on the daily GP data collection comprising over 900 checks covering the integrity, structure and format of the data.[25]

Recorded information in both datasets includes patients' demography, clinical symptoms, signs, investigations, diagnoses, prescriptions, referrals, behavioural factors and test results entered by clinicians and other practice staff. All clinical information is coded using Read Codes in CPRD-GOLD and SNOMED clinical terms (CT) in CPRD-AURUM.[26 27] In the proposed study, extracted patients will have patient-level data linked to Hospital Episode Statistics (HES) Admitted Patient Care (APC) and Diagnostic Imaging Dataset (DID), Office for National statistics (ONS) Death Registration, patient-level deprivation and practice-level deprivation to provide a more comprehensive dataset. The CPRD dataset has been used to develop or validate a range of risk prediction models, including in cardiovascular disease.[19 28]

The extracted datasets, including linked data, comprise all patients for the period between 2nd January 1998 and 30th November 2018 from the snapshot of CPRD-GOLD and CPRD-AURUM provided in October 2019. Over this study period, the CPRD-GOLD dataset comprises approximately 4.5 million patients eligible for data linkage at an up-to-standard practice, with over 200,000 patients having a record of AF. The CPRD-AURUM dataset comprises approximately 18 million patients eligible for data linkage, with almost 800,000 patients having a record of AF. A sample of 245 general practices will be randomly selected from 800 general practices in CPRD-AURUM to approximately match the size of CPRD-GOLD.

Patient and Public Involvement

Patients and public were not involved in the design of this research. However, a Scientific Advisory Board, including representatives from the Arrhythmia Alliance, National Institute for Health and Care Excellence (NICE) atrial fibrillation guideline committee lay members (last updated 04/01/2020) and EHR software providers, has been created to provide expert

For peer review only

context advice on the research, the dissemination of results and advise on the translation of the findings of this study into clinical practice.

Inclusion and Exclusion Criteria

The study population will comprise all available patients in CPRD-GOLD and CPRD-AURUM eligible for data linkage and with at least 1-year follow-up in the period between 2nd January 1998 and 30th November 2018. Patients will be excluded if they were 18 years of age or under at the date of the first registration in CPRD, diagnosed with AF or atrial flutter (AFl) before 1st January 1998, registered for less than 1 year in CPRD or ineligible for data linkage.

Outcome Ascertainment

The outcome of interest is first diagnosed AF or AFl after baseline (1st January 2009). We have included AFl as an outcome since it has similar clinical relevance, including thromboembolic risk and anticoagulation guidelines, as AF.[1] These will be identified using Read codes and SNOMED CT in CPRD datasets. For HES APC events and underlying cause of death variable in the ONS Death Registration data file, the tenth revision of the International Statistical Classification of Diseases and Related Health Problems (ICD-10) codes will be used. Misclassified data can lead to systematic prediction errors and accuracy of data may vary over time.[29] CPRD has converted older ICD codes to the newer version, increasing confidence in their validity. Nonetheless, to verify data accuracy we will check the data accuracy by year and include the year of AF diagnosis in the prediction models to assess their impact.

Sample size

To develop a prognostic prediction model, the required sample size may be determined by three criteria suggested by *Riley et al*.[30] For example, suppose a maximum of 200 parameters will be included in the prediction model and the Cox-Snell generalised R^2 is assumed to be 0.01. A total of 377,996 patients will be required to meet Riley's criterion (i) with global shrinkage factor of 0.95; this sample size also ensures a small absolute difference ($\Delta < 0.05$) in the apparent and adjusted Nagelkerke R^2 (Riley's criterion (ii)) and ensures precise estimate of overall risk with a margin of error < 0.001 (Riley's criterion (iii)). According to the Quality and Outcomes Framework (QOF), the prevalence of AF in England is 1.7%.[31 32] Given an AF prevalence of 1.7%, only 6,425 patients will be expected to develop AF from 377,996 patients. Therefore, the number of patients in the CPRD dataset with AF will provide sufficient statistical power to develop and validate a deep learning prediction algorithm with the predefined precision and accuracy.

Predictor Variables

A systematic review has highlighted 22 predictor variables included in varying combinations by 10 preceding prediction models developed to detect incident AF in the community.[16] In addition, a more recently-published machine learning model, has established six further time-varying variables (e.g. change in body mass index between the latest two quarters).[19]

For peer review only - http://bmjopen.bmj.com/site/about/guidelines.xhtml

To capture the full potential of deep learning and this large dataset, we will broaden our search for candidate predictors to all available variables, whilst retaining temporal information (including all clinical assessments, hospitalised events and medications). The potential predictors may include:

1. Sociodemographic variables including age, sex, ethnicity and indices of multiple deprivation.
2. All disease conditions during follow-up, including hospitalised diseases and procedures, such as other cardiovascular diseases, diabetes mellitus, chronic lung disease, renal disease, inflammatory disease, cancer, hypothyroidism and surgical procedures.
3. Clinical assessments including heart rate, systolic and diastolic blood pressure, height, weight and body mass index.
4. Medications prescribed including antihypertensives, statins, antidepressants, anxiolytics/hypnotics and antipsychotics.
5. Lifestyle factors including smoking status and alcohol consumption.
6. All biomarkers routinely collected during follow-up including total, high-density lipoprotein and low-density lipoprotein cholesterol, triglycerides, creatinine, c-reactive protein, erythrocyte sedimentation rate.

Predictive factors will be identified using the appropriate codes. In CPRD, we will use Read codes for diagnoses, measurements (e.g. systolic and diastolic values) and product codes (prodcodes) for medications. In HES APC, we will use ICD-10 codes and Office of Population Censuses and Surveys Classification of Interventions and Procedures version 4 (OPCS-4) codes. In the ONS Death Registration data file, we will use ICD-10 codes (and ICD-9 codes for the period before 2001).

Missing data

Missing data is expected in EHR data, and will be handled using multiple imputations according to the approaches suggested by Carpenter and Kenward, and depending on the amount of missing data.[33] It is likely there will be misclassification of baseline characteristics, such as smoking status, cholesterol, blood pressure and weight that change over time. To account for this, we will define baseline information using only measures recorded within the last year. We will then use measures taken within the last 2-5 years and in the year after baseline as parameters in the chained imputation equations used to impute baseline covariates with ≤ 40% missingness. In addition, median imputation, a common approach to deal with missing values in machine learning algorithms will be used to test the model robustness.[34]

Data analysis plan

Development and external validation of model

The CPRD-GOLD and CPRD-AURUM data will be cleaned and preprocessed for model development and validation, respectively. Specifically, for patient features with binary values, 0 and 1 will be mapped to the binary values. Variables with multiple categories will be split into their component categories, and each given a binary value to indicate the

For peer review only - http://bmjopen.bmj.com/site/about/guidelines.xhtml

presence or not of the variable for each patient. Continuous variables will be kept as continuous. To reduce the high cardinality of Read codes and ICD codes we will map both to Caliber codes,[35] which is an expert checked mapping directory from University College London. Prodcodes can be mapped to level 2 of BNF (British National Formulary) codes.[36]

A number of deep learning models will be investigated for prediction of AF in CPRD-GOLD. These will include a convolutional neural network (CNN), a recurrent neural network (RNN) and a Transformer architecture. They each possess characteristics that can capture the temporality of EHR data and the progression of a patient's health status.

Although CNNs are typically associated with static content (e.g. images and documents) they are increasingly used to uncover temporal relationships in EHR.[37-40] The patient EHR can be converted to a temporal matrix, as proposed in *Cheng et al.* with one dimension corresponding to time and the other dimension corresponding to medical events (Figure 1a).[37] A one-sided convolution operation can be applied to each possible window of features in the event matrix to produce a feature map followed with max-pooling (to capture the most important features) and culminating in a fully connected layer and softmax classifier. If the time dimension is embedded in 1 day increments, we have the option to learn temporal features by extending connectivity in the time dimension through a range of fusion strategies. Temporal early fusion could combine information across an entire time window to establish global patterns, temporal late fusion can be performed in the fully connected layer to model local connections, and temporal slow fusion could capture both by extending the connectivity of all convolutional layers.[37]

Patient EHR events including diagnoses, procedures and medications can also be represented as a sequence of codes over time, similar to words in a sentence (Figure 1b). After embedding into a lower dimensional space a one-dimensional convolutional operation over the temporal dimension with a combination of filters of different lengths could capture temporal dependencies at multiple levels as proposed in *Che et al*.[38]

A sequence-based representation also makes patient EHR amenable to techniques that have provided breakthroughs in natural language processing, especially RNNs.[41-43] Recurrent neural networks with a gated recurrent unit (GRU) model design have been successfully applied in modelling sequential structured EHR data to predict diagnoses as they can accept an input vector at each time step while storing information in a hidden layer which changes over time.[21 44-51] We will apply the reverse time attention model (RETAIN), which has been tested for the prediction of heart failure in CPRD.[52] RETAIN can generate temporal attention vectors at both visit and variable-level by running RNNs backwards, which somewhat mimics a physician's behaviours in examining a patient's past visits in reverse time order and could capture the short-term time-varying events that were found to improve prediction in a previous shallow neural network model.[19 46] Timestamps can be included to calculate the attentions for the entire visit sequence and thus provide temporal information for each visit.[46]

Finally, Transformers have become state-of-art in natural language processing,[53 54] and we will apply an adaption for EHR, BEHRT (BERT for EHR), which was originally developed in CPRD.[55] This model's structure, depicting each diagnosis code as a word, with each visit as a sentence and the entire medical history as a document facilitates multi-head self-attention, positional encoding and masked language model for EHR. BEHRT can define a representation incorporating information on the diseases afflicting a patient, the positional interactions of diseases and sequence of events, with the age of a patient linked to each visit

For peer review only

to give a sense of time between diagnoses.[55] This representation can then be used for a number of tasks including disease prediction.

Preprocessed patient-level data in CPRD-GOLD will be randomly split into an 80:20 ratio to create training and testing samples. The split ratio is not a significant factor, given the volume of the sample size. The model parameters and dropout rate, will be chosen through a grid search and 10-fold cross-validation will be used (i.e. 10% of the training data will be randomly selected as the cross-validation set).

Whilst the outcome is binary, instead of just predicting 0 or 1 for a patient (new AF or not), the probability of that patient developing AF by different time points over months (1-, 3-, 6-) and years (1-, 5-, 10-) msy be predicted. The probability of developing AF at each time point could be plotted to give an understanding of evolving risk of AF. The clinical risk prediction performance of the deep learning models will be compared against a range of classic machine learning techniques and traditional statistical predictive modelling methods including support vector machine (SVM), random forest (RF), naïve Bayes (NB) and Cox proportional hazards model (CPH). Discrimination (Harrell's c-statistic and area under receiver operating characteristics) and calibration metrics will be supplemented with positive and negative predictive values, precision, recall and area under precision-recall curve for all models.

The CPRD-AURUM dataset will then externally validate the model performance to assess generalisability. It has been shown that a lack of external validation has hampered the implementation of previous machine learning models in routine clinical practice.[56]

Identification and quantification of the magnitude of predictors

The proposed deep learning models can extract informative risk factors from EHR data. Specifically, a risk factor selection strategy proposed in *Huang et al.* will be adapted to identify informative risk factors.[57] The model will provide weights of the identified risk factors to help understand the significance of risk factors at different risk levels. The impact of the number of risk factors on AF risk prediction performance will be assessed through the curves of both AUC and prediction accuracy plotted against the number of risk factors. Some predictors, such as body mass index, blood pressure, frequency of visits and strength of the prescribed medication, may change over time. The incremental prognostic values of including these variable trajectories will be explored, and the impact on predictive accuracy will be assessed.

Software

The deep learning model will be implemented in R (through the R studio interface) and TensorFlow or Python and PyTorch including data preprocessing, missing data imputation, model development and validation.

For peer review only - http://bmjopen.bmj.com/site/about/guidelines.xhtml

ETHICS AND DISSEMINATION

The study has been approved by CPRD (ref no: 19_076). Those handling data have completed University of Leeds information security training. All analyses will be conducted in concordance with the CPRD study dataset agreement between the Secretary of State for Health and Social Care and the University of Leeds. The study has been registered at clinical trials.gov (NCT04657900).

The study is informed by the Prognosis Research Strategy (PROGRESS) framework and recommendations.[58] The subsequent research paper will be submitted for publication in a peer-reviewed journal and will be written following TRIPOD: *transparent reporting of a multivariable prediction model for individual prognosis or diagnosis* and RECORD: *reporting of studies conducted using observational routinely-collected health data* guidelines.[59 60]

If the model succeeds (defined as improving predicting accuracy by at least 5% compared with existing models), the algorithm could be made readily available through free-to-use software. The model will be designed to be amenable to in-situ updating with new information so that prediction of an individual's AF risk is updated contemporaneously. The algorithm could be a built-in tool for use in general practices to 'screen' for patients at high risk of developing new onset AF. Future research will be needed to assess the clinical impact of this risk model. At the point when utilisation in clinical practice is possible the applicable regulation on medicine devices will be adhered to.[61] When in clinical use, the model itself could also be reviewed and updated by a pre-specified expert consensus group on an annual basis after incorporating evidence from post-service utilization and the curation of more data.

For peer review only

CONCLUSIONS

Atrial Fibrillation is a common clinical problem with potentially catastrophic sequelae. A prediction model that may identify in a community setting which individuals will develop AF, and when this is most likely to occur, could enable targeted screening. This British Heart Foundation funded study will contribute to knowledge about the detection of AF through prediction using a data science approach in routine EHR data. The use of artificial intelligence technology may uncover new predictors in EHR and facilitate easier translation into clinical practice.

For peer review only

Authors' contributions

JW and CPG conceived the concept and JW planned the analysis. RN wrote the first draft, with contributions from all authors. All authors (RN, JW, DH, AF, CC, CPG) approved the final version and jointly take responsibility for the decision to submit the manuscript to be considered for publication.

Funding statement

RN is supported by the British Heart Foundation Clinical Research Training Fellowship (FS/20/12/34789).

Competing Interests

None declared

For peer review only

REFERENCES

1. Hindricks G, Potpara T, Dagres N, et al. 2020 ESC Guidelines for the diagnosis and management of atrial fibrillation developed in collaboration with the European Association of Cardio-Thoracic Surgery (EACTS). *Eur Heart J* 2020 doi: 10.1093/eurheartj/ehaa612 [published Online First: 2020/08/30]
2. Benjamin EJ, Virani SS, Callaway CW, et al. American heart association council on epidemiology and prevention statistics committee and stroke statistics subcommittee. *Heart disease and stroke statistics-2018 update: a report from the American Heart Association Circulation* 2018;137(12):e67-e492.
3. Ruff CT, Giugliano RP, Braunwald E, et al. Comparison of the efficacy and safety of new oral anticoagulants with warfarin in patients with atrial fibrillation: a meta-analysis of randomised trials. *Lancet* 2014;383(9921):955-62. doi: 10.1016/S0140-6736(13)62343-0 [published Online First: 2013/12/10]
4. Freedman B, Camm J, Calkins H, et al. Screening for atrial fibrillation: a report of the AF-SCREEN International Collaboration. *Circulation* 2017;135(19):1851-67.
5. Boriani G, Laroche C, Diemberger I, et al. Asymptomatic atrial fibrillation: clinical correlates, management, and outcomes in the EORP-AF Pilot General Registry. *Am J Med* 2015;128(5):509-18 e2. doi: 10.1016/j.amjmed.2014.11.026 [published Online First: 2014/12/24]
6. Aronsson M, Svennberg E, Rosenqvist M, et al. Cost-effectiveness of mass screening for untreated atrial fibrillation using intermittent ECG recording. *Ep Europace* 2015;17(7):1023-29.
7. Hobbs FR, Fitzmaurice D, Mant J, et al. A randomised controlled trial and cost-effectiveness study of systematic screening (targeted and total population screening) versus routine practice for the detection of atrial fibrillation in people aged 65 and over. The SAFE study. 2005
8. Svennberg E, Engdahl J, Al-Khalili F, et al. Mass screening for untreated atrial fibrillation: the STROKESTOP study. 2015;131(25):2176-84.
9. Committee UNS. The UK NSC recommendation on Atrial Fibrillation screening in adults 2019 [cited 2021 April 26]. Available from: https://legacyscreening.phe.org.uk/atrialfibrillation#:~:text=The%20UK%20NSC%20does%20not,in%20people%20found%20through%20screening.
10. Moons KG, Kengne AP, Woodward M, et al. Risk prediction models: I. Development, internal validation, and assessing the incremental value of a new (bio) marker. *Heart* 2012;98(9):683-90.
11. Attia ZI, Noseworthy PA, Lopez-Jimenez F, et al. An artificial intelligence-enabled ECG algorithm for the identification of patients with atrial fibrillation during sinus rhythm: a retrospective analysis of outcome prediction. *The Lancet* 2019;394(10201):861-67.
12. Alonso A, Krijthe BP, Aspelund T, et al. Simple risk model predicts incidence of atrial fibrillation in a racially and geographically diverse population: the CHARGE-AF consortium. *J Am Heart Assoc* 2013;2(2):e000102. doi: 10.1161/JAHA.112.000102 [published Online First: 2013/03/30]
13. Schnabel RB, Sullivan LM, Levy D, et al. Development of a risk score for atrial fibrillation (Framingham Heart Study): a community-based cohort study. *The Lancet* 2009;373(9665):739-45. doi: 10.1016/s0140-6736(09)60443-8

14. Aronson D, Shalev V, Katz R, et al. Risk Score for Prediction of 10-Year Atrial Fibrillation: A Community-Based Study. *Thromb Haemost* 2018;118(9):1556-63. doi: 10.1055/s-0038-1668522 [published Online First: 2018/08/14]
15. Li YG, Pastori D, Farcomeni A, et al. A Simple Clinical Risk Score (C2HEST) for Predicting Incident Atrial Fibrillation in Asian Subjects: Derivation in 471,446 Chinese Subjects, With Internal Validation and External Application in 451,199 Korean Subjects. *Chest* 2019;155(3):510-18. doi: 10.1016/j.chest.2018.09.011 [published Online First: 2018/10/08]
16. Himmelreich JC, Veelers L, Lucassen WA, et al. Prediction models for atrial fibrillation applicable in the community: a systematic review and meta-analysis. *EP Europace* 2020;22(5):684-94.
17. Himmelreich JC, Lucassen WA, Harskamp RE, et al. CHARGE-AF in a national routine primary care electronic health records database in the Netherlands: validation for 5-year risk of atrial fibrillation and implications for patient selection in atrial fibrillation screening. 2021;8(1):e001459.
18. Kolek MJ, Graves AJ, Xu M, et al. Evaluation of a prediction model for the development of atrial fibrillation in a repository of electronic medical records. *JAMA cardiology* 2016;1(9):1007-13.
19. Hill NR, Ayoubkhani D, McEwan P, et al. Predicting atrial fibrillation in primary care using machine learning. *PLoS One* 2019;14(11):e0224582.
20. Weng SF, Reps J, Kai J, et al. Can machine-learning improve cardiovascular risk prediction using routine clinical data? *PLoS One* 2017;12(4):e0174944.
21. Health-atm: A deep architecture for multifaceted patient health record representation and risk prediction. Proceedings of the 2018 SIAM International Conference on Data Mining; 2018. SIAM.
22. Chen D, Liu S, Kingsbury P, et al. Deep learning and alternative learning strategies for retrospective real-world clinical data. 2019;2(1):1-5.
23. Herrett E, Gallagher AM, Bhaskaran K, et al. Data resource profile: clinical practice research datalink (CPRD). *Int J Epidemiol* 2015;44(3):827-36.
24. Herrett E, Thomas SL, Schoonen WM, et al. Validation and validity of diagnoses in the General Practice Research Database: a systematic review. *Br J Clin Pharmacol* 2010;69(1):4-14.
25. Wolf A, Dedman D, Campbell J, et al. Data resource profile: Clinical practice research datalink (cprd) aurum. *Int J Epidemiol* 2019;48(6):1740-40g.
26. Chisholm J. The Read clinical classification. *BMJ: British Medical Journal* 1990;300(6732):1092.
27. SNOMED clinical terms: overview of the development process and project status. Proc AMIA Symp; 2001. American Medical Informatics Association.
28. Hippisley-Cox J, Coupland C, Brindle P. Development and validation of QRISK3 risk prediction algorithms to estimate future risk of cardiovascular disease: prospective cohort study. *BMJ* 2017;357:j2099.
29. Ehrenstein V, Nielsen H, Pedersen AB, et al. Clinical epidemiology in the era of big data: new opportunities, familiar challenges. *Clin Epidemiol* 2017;9:245.
30. Riley RD, Snell KI, Ensor J, et al. Minimum sample size for developing a multivariable prediction model: PART II-binary and time-to-event outcomes. *Stat Med* 2019;38(7):1276-96.

For peer review only

31. Cowan JC, Wu J, Hall M, et al. A 10 year study of hospitalized atrial fibrillation-related stroke in England and its association with uptake of oral anticoagulation. *Eur Heart J* 2018;39(32):2975-83.
32. Wu J, Alsaeed ES, Barrett J, et al. Prescription of oral anticoagulants and antiplatelets for stroke prophylaxis in atrial fibrillation: nationwide time series ecological analysis. *EP Europace* 2020;22(9):1311-19.
33. Carpenter J, Kenward M. Multiple imputation and its application: John Wiley & Sons 2012.
34. Batista GE, Monard MC. An analysis of four missing data treatment methods for supervised learning. *Applied artificial intelligence* 2003;17(5-6):519-33.
35. Kuan V, Denaxas S, Gonzalez-Izquierdo A, et al. A chronological map of 308 physical and mental health conditions from 4 million individuals in the English National Health Service. 2019;1(2):e63-e77.
36. BNF Publications [22 April 2021]. Available from: https://www.bnf.org/.
37. Risk prediction with electronic health records: A deep learning approach. Proceedings of the 2016 SIAM International Conference on Data Mining; 2016. SIAM.
38. Che Z, Cheng Y, Sun Z, et al. Exploiting convolutional neural network for risk prediction with medical feature embedding. 2017
39. Development of Deep Learning Algorithm for Detection of Colorectal Cancer in EHR Data. Medinfo; 2019.
40. Suo Q, Ma F, Yuan Y, et al. Deep patient similarity learning for personalized healthcare. 2018;17(3):219-27.
41. Mikolov T, Sutskever I, Chen K, et al. Distributed representations of words and phrases and their compositionality. 2013
42. Zaremba W, Sutskever I, Vinyals OJapa. Recurrent neural network regularization. 2014
43. Shickel B, Tighe PJ, Bihorac A, et al. Deep EHR: a survey of recent advances in deep learning techniques for electronic health record (EHR) analysis. 2017;22(5):1589-604.
44. Doctor ai: Predicting clinical events via recurrent neural networks. Machine learning for healthcare conference; 2016. PMLR.
45. Choi E, Schuetz A, Stewart WF, et al. Using recurrent neural network models for early detection of heart failure onset. 2017;24(2):361-70.
46. Choi E, Bahadori MT, Kulas JA, et al. Retain: An interpretable predictive model for healthcare using reverse time attention mechanism. 2016
47. GRAM: graph-based attention model for healthcare representation learning. Proceedings of the 23rd ACM SIGKDD international conference on knowledge discovery and data mining; 2017.
48. Dipole: Diagnosis prediction in healthcare via attention-based bidirectional recurrent neural networks. Proceedings of the 23rd ACM SIGKDD international conference on knowledge discovery and data mining; 2017.
49. Kwon BC, Choi M-J, Kim JT, et al. Retainvis: Visual analytics with interpretable and interactive recurrent neural networks on electronic medical records. 2018;25(1):299-309.
50. Kame: Knowledge-based attention model for diagnosis prediction in healthcare. Proceedings of the 27th ACM International Conference on Information and Knowledge Management; 2018.
51. Choi E, Xiao C, Stewart WF, et al. Mime: Multilevel medical embedding of electronic health records for predictive healthcare. 2018

For peer review only

52. Solares JRA, Raimondi FED, Zhu Y, et al. Deep learning for electronic health records: A comparative review of multiple deep neural architectures. 2020;101:103337.
53. Devlin J, Chang M-W, Lee K, et al. Bert: Pre-training of deep bidirectional transformers for language understanding. 2018
54. Vaswani A, Shazeer N, Parmar N, et al. Attention is all you need. 2017
55. Li Y, Rao S, Solares JRA, et al. BEHRT: transformer for electronic health records. 2020;10(1):1-12.
56. Banerjee A, Chen S, Fatemifar G, et al. Machine learning for subtype definition and risk prediction in heart failure, acute coronary syndromes and atrial fibrillation: systematic review of validity and clinical utility. 2021;19(1):1-14.
57. Huang Z, Dong W, Duan H, et al. A regularized deep learning approach for clinical risk prediction of acute coronary syndrome using electronic health records. *IEEE Trans Biomed Eng* 2017;65(5):956-68.
58. Steyerberg EW, Moons KG, van der Windt DA, et al. Prognosis Research Strategy (PROGRESS) 3: prognostic model research. *PLoS Med* 2013;10(2):e1001381.
59. Collins GS, Reitsma JB, Altman DG, et al. Transparent Reporting of a Multivariable Prediction Model for Individual Prognosis or Diagnosis (TRIPOD) The TRIPOD Statement. *Circulation* 2015;131(2):211-19.
60. Nicholls SG, Quach P, von Elm E, et al. The reporting of studies conducted using observational routinely-collected health data (RECORD) statement: methods for arriving at consensus and developing reporting guidelines. *PLoS One* 2015;10(5):e0125620.
61. Kramer DB, Xu S, Kesselheim AS. Regulation of medical devices in the United States and European Union. The Ethical Challenges of Emerging Medical Technologies: Taylor and Francis 2020:41-49.

For peer review only

Patient consent for publication
Not required

Ethics Approval
Permissions for the CPRD-GOLD and CPRD-AURUM datasets were obtained from CPRD (ref no: 19_076). The study was approved by CPRD ethical approval committee.

Word Count
3603

Figure Legend
Figure 1: An example of how a patient's EHR could be represented as a temporal matrix (A) compared to a sequence (B). In (A) time is on the x dimension and binary medical events are on the y dimension. In (B) the temporal information, in this example, is represented as inter-visit interval through timestamps (e.g. t2 –t1)

For peer review only

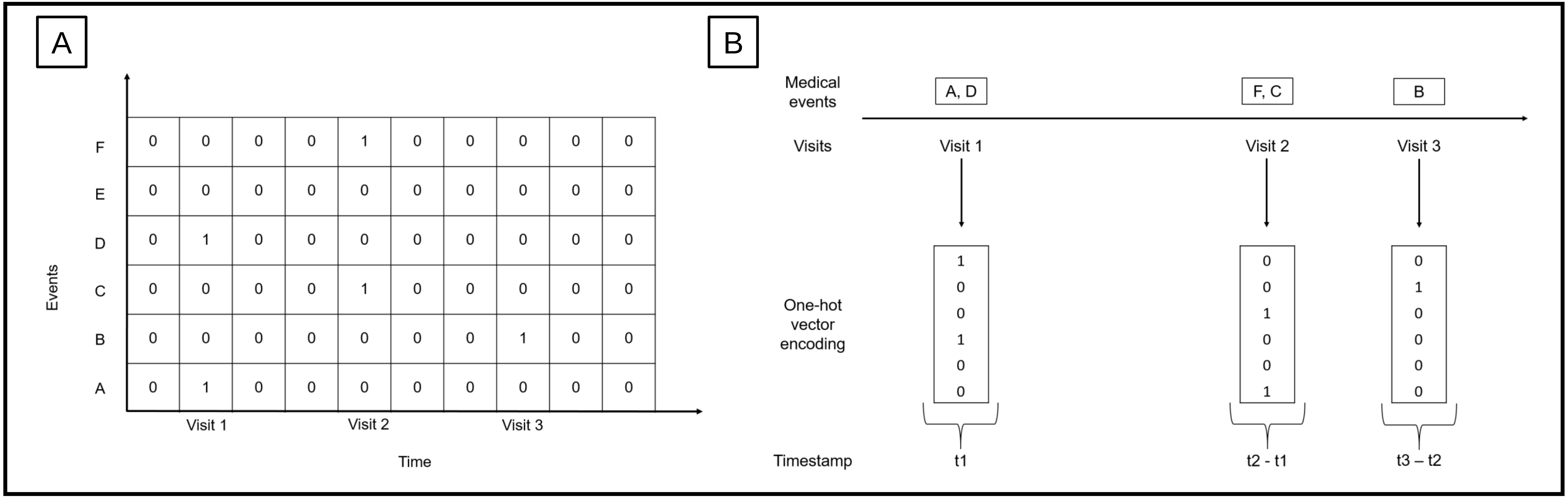

Figure 1 An example of how a patient's EHR could be represented as a temporal matrix (A) compared to a sequence (B). In (A) time is on the x dimension and binary medical events are on the y dimension. In (B) the temporal information, in this example, is represented as inter-visit interval through timestamps (e.g. t2 – t1)
